# Small volume bone marrow aspirates with high progenitor cell concentrations maximize cell therapy dose manufacture and substantially reduce donor hemoglobin loss

**DOI:** 10.1186/s12916-023-03059-3

**Published:** 2023-09-19

**Authors:** Jeremy Epah, Gabriele Spohn, Kathrin Preiß, Markus M. Müller, Johanna Dörr, Rainer Bauer, Shabnam Daqiq-Mirdad, Joachim Schwäble, Stefanie N. Bernas, Alexander H. Schmidt, Erhard Seifried, Richard Schäfer

**Affiliations:** 1 Institute for Transfusion Medicine and Immunohaematology, German Red Cross Blood Donor Service Baden-Württemberg-Hessen gGmbH, Goethe University Hospital, Frankfurt Am Main, Germany; 2 DKMS, Tübingen, Germany; 3 Institute for Transfusion Medicine and Gene Therapy, Medical Center, University of Freiburg, Hugstetter Str. 55, 79106 Freiburg, Germany

**Keywords:** Bone marrow, Collection, Transplantation, Stem cells, Anemia, MSCs

## Abstract

**Background:**

Bone marrow (BM) transplantation is a life-saving therapy for hematological diseases, and the BM harbors also highly useful (progenitor) cell types for novel cell therapies manufacture. Yet, the BM collection technique is not standardized.

**Methods:**

Benchmarking our collection efficiency to BM collections worldwide (*N* = 1248), we noted a great variability of total nucleated cell (TNC) yields in BM products (HPC-M) with superior performance of our center, where we have implemented a small volume aspirate policy. Thus, we next prospectively aimed to assess the impact of BM collection technique on HPC-M quality. For each BM collection (*N* = 20 donors), small volume (3 mL) and large volume (10 mL) BM aspirates were sampled at 3 time points and analyzed for cell composition.

**Results:**

Compared to large volume aspirates, small volume aspirates concentrated more TNCs, immune cells, platelets, hematopoietic stem/progenitor cells, mesenchymal stromal cells (MSCs), and endothelial progenitors. Inversely, the hemoglobin concentration was higher in large volume aspirates indicating more hemoglobin loss. Manufacturing and dosing scenarios showed that small volume aspirates save up to 42% BM volume and 44% hemoglobin for HPC-M donors. Moreover, MSC production efficiency can be increased by more than 150%.

**Conclusions:**

We propose to consider small volume BM aspiration as standard technique for BM collection.

**Supplementary Information:**

The online version contains supplementary material available at 10.1186/s12916-023-03059-3.

## Background

The practice of hematopoietic stem cell transplantation (HSCT) has long been established as state-of-the-art life-saving therapy for hematological diseases ranging from malignancies to immunological disorders and inborn errors of metabolism [1, 2]. The advent of mobilization agents, such as granulocyte-colony stimulating factor or AMD3100, enabled, together with advanced apheresis technologies, the efficient collection of hematopoietic stem/progenitor cells (HSPCs) from the peripheral blood (PBSC) [3]. Yet, bone marrow (BM) is still the preferred cell source for specific disease indications in adults (e.g., aplastic anemia) and for the majority of pediatric allogeneic HSCTs [4, 5]. Meanwhile, BM is coming increasingly into focus for haplo-identical HSCT and as an important source for novel cell therapies such as mesenchymal stromal cells (MSCs), featuring potent immunomodulatory effects in graft-versus-host disease (GvHD) [6, 7]. Additionally, BM can, to a lesser extent than PBSC, be used as starting material for gene therapies manufacture [8].

However, BM is a scarce resource and its collection in the allogeneic setting is not in the interest of the donor’s own physical health. Performed since decades, the procedure has generally proved being safe, but BM donors have a threefold higher risk for life-threatening, serious adverse events compared to PBSC donors leading to a higher rate of unplanned hospitalizations [9].

Such, ensuring donor safety, while providing a sufficient total nucleated cell (TNC) transplantation dose, minimally 2 × 10^8^ viable TNCs per kilogram patient’s body weight (BW), is a mandatory, but sometimes delicate, balance [10]. The collected BM volume shall not exceed 20 mL/kg donor’s BW and could make up to 25% of the total blood volume [11]. Therefore, reaching the desired TNC yield can be particularly challenging with low donor and high patient BW and/or when exceptionally high TNC numbers are requested.

This problem can even be aggravated when collection techniques are used that may lead to significant “dilution” of the BM with peripheral blood (PB) [11]. Specifically, a substantial fraction of PB in the BM aspirate may not only decrease the concentration, and such the maximum content, of nucleated cells in the BM product (HPC-M) but also lowers the donor’s hemoglobin (Hb) level significantly [12]. This can negatively affect the donor’s well-being, e.g., by prolonged weakness and fatigue [12, 13]. In some cases, this can be so severe that centers opt for using preoperatively collected autologous blood to compensate the post-harvest Hb loss [12, 14].

Surprisingly, despite the clinical significance of BM harvesting and the outlined challenges, the technique for collecting BM is not standardized. Thus, varying results in TNC collection efficiency for HPC-M manufacture between centers are reported consistently [15]. We found higher TNC yields in the HPC-M products collected at our center (German Red Cross Blood Donor Service Baden-Württemberg-Hessen gGmbH, Goethe University Hospital, Frankfurt, Germany) compared to other centers worldwide. We have implemented a small BM aspirate volume policy, and, therefore, we hypothesized that small volume BM aspirates are higher concentrated for nucleated cells, whereas larger BM aspirates are more diluted with PB, hereby collecting fewer nucleated cells per milliliter. Hence, we then prospectively assessed the impact of different BM collection techniques on the quality of HPC-M products. Specifically, we obtained small and large volume aspirates at three different time points during each BM collection and analyzed their respective cell compositions in detail. Such, we could not only verify the first part of our hypothesis, but we also provide evidence that the lower concentrations of TNCs, HSPCs, and other cell types in large volume BM aspirates are not the mere result of dilution with PB but related to the specific gravity of nucleated cells and red blood cells (RBCs). Eventually, applying TNC and CD34pos target dose and MSC dose manufacturing projection models, we show that manufacturing HPC-M products with small volume BM aspirates substantially reduce the required BM volume, increase cell therapies’ manufacture efficiency, and minimize Hb loss for donors.

## Methods

### Bone marrow products data

DKMS [16, 17] is a major international stem cell donor center with 12 million registered donors in 7 countries (Chile, Germany, India, Poland, South Africa, the UK, the USA). Since 1991, donors registered with DKMS have donated stem cells (BM or PBSC) 111,000 times, including 7705 times in 2022. In that year, DKMS donors contributed 35.4% (7705/21,767) of all unrelated stem cell products worldwide [18]. DKMS receives an individual collection report following each stem cell collection of a DKMS donor. This report includes TNC count and concentration, and the ratio of collected to requested TNCs. Reports were analyzed for BM collections performed in Germany (GER; *N* = 910, of which 81 were performed at our center), Poland (PL; *N* = 209), the USA (USA; *N* = 96), and the UK (UK; *N* = 33) in 2019. Overall, 25 collections were excluded from the analysis (21 from GER, 2 from UK, 1 from PL, 1 from USA) since relevant information was missing on the collection report.

### Bone marrow collection

The study was approved by the ethics committee of the Goethe University Hospital, Frankfurt, Germany (approval #19–286).

Human BM samples were obtained after informed donor consent from 20 healthy, adult allogeneic donors (4 female and 16 male, aged 18–56 [mean: 29.6] years; Table 1) during BM collections for clinical HPC-M products using a Jamshidi needle (SELECTIVE “ILY” type, ZAMAR®, Vrsar, Croatia). Small volume (3 mL) and large volume (10 mL) aspirates, anticoagulated with heparin (Li-Heparin Vacutainer, Becton–Dickinson, Heidelberg, Germany), were sampled at the start (t1), at halftime (t2), and at the end (t3) of each BM collection. To standardize sampling and to exclude possible impact of the needle position in the punction canal, each sample was taken from separate punctions as first aspirate after reaching the BM cavity. In detail, a 3-mL BM aspirate was sampled immediately (first aspirate) after the first puncture of the iliac crest at t1 (puncture A). The following aspirates of this puncture channel were used for the HPC-M product. In a second, independent, puncture location at t1, a 10-mL BM aspirate was collected as first aspirate of this channel (puncture B), and the following aspirates of this channel were used for the product. This was repeated at t2 (punctures C and D) and at t3 (punctures E and F) (Additional file 1: Fig. S1). To minimize possible operator effects, six operators in total performed BM sampling. Thereby, different individuals took the small and large volume samples randomly. For calculation of cell concentrations, exact volumes of the BM samples were recorded.

**Table 1:** Data from 20 healthy, adult allogeneic donors

Donor	Sex	Age
1	Male	28
2	Female	27
3	Male	27
4	Male	29
5	Female	27
6	Male	18
7	Male	22
8	Male	37
9	Male	25
10	Male	21
11	Male	27
12	Female	25
13	Female	56
14	Male	35
15	Male	30
16	Male	30
17	Male	36
18	Male	28
19	Male	36
20	Male	28

### BM processing

After dilution with DPBS (Thermo Fisher Scientific, Darmstadt, Germany) and filtering (CellStrainer, Corning, Amsterdam, The Netherlands), TNC counts, hemoglobin content, hematocrit, RBC, and platelet counts were determined using the Sysmex XN-350 automatic hemocytometer (Sysmex Deutschland GmbH, Norderstedt, Germany). The BM aspirates were further processed by density gradient centrifugation on lymphocyte separation medium (Lonza, Basel, Switzerland). Cell pellets were resuspended in DPBS and TNC counts determined using Sysmex XN-350.

### Flow cytometry/immunophenotype/cell composition

Flow cytometry was performed with LSRFortessa (Becton–Dickinson). In detail, 2 × 10^6^ cells each were stained in three different multicolor panels, comprised of markers for HSPCs and endothelial progenitor cells (EPCs), immune cell subsets and MSCs and their precursors, followed by RBC lysis (BD Pharm Lyse™ Lysing Buffer, BD Biosciences, Heidelberg, Germany).

For detection and quantification of HSPCs and EPCs, cells were stained with 7-AAD viability dye (BioLegend, San Diego, CA, USA) and the following antibodies (all from BioLegend, unless otherwise noted): anti-CD14-APC-Cy7 (63D3), anti-CD31-Brilliant Violet 605™ (WM59), anti-CD34-Alexa Fluor® 700 (581), anti-CD45-V500 (HI30, BD Horizon™), anti-CD117-Alexa Fluor® 488 (104D2), anti-CD133-Brilliant Violet 421™ (clone 7), and anti-CD309-PE (7D4-6).

For analysis of immune cell subsets, cells were stained with 7-AAD viability dye and the following antibodies (all from BioLegend): anti-CD3-Pacific Blue (HIT3a), anti-CD4-Alexa Fluor® 700 (SK3), anti-CD8-Brilliant Violet 510™ (SK1), anti-CD11b-PE-Cy7 (LM2), anti-CD14-APC-Cy7 (63D3), anti-CD19-PE (HIB19), anti-CD45-FITC (HI30), anti-CD56-Alexa Fluor® 647 (5.1H11), anti-CD183-Brilliant Violet 605™ (G025H7), and anti-CD194-PE-Dazzle™ 594 (L291H4).

MSCs and their precursors were detected in the BM aspirates by staining with 7-AAD viability dye and the following antibodies (all from BioLegend, unless otherwise noted): anti-CD29-APC-Cy7 (TS2/16), anti-CD34-Alexa Fluor® 700 (581), anti-CD45-FITC (HI30), anti-CD73-Brilliant Violet 605™ (AD2), anti-CD90-PE-Cy7 (5E10, BD Horizon™), anti-CD105-PE-CF594 (266, BD Horizon™), anti-CD119-PE (GIR-208), anti-CD146-Brilliant Violet 510™ (P1H12), and anti-CD271-BV421 (C40-1457, BD Horizon™).

Flow cytometry data were analyzed using FCS Express 6 Flow Software (De Novo Software, Pasadena, CA, USA). The percentages of viable cells of each cell type were converted to cell count per milliliter processed BM.

### Colony-forming unit cell assay

To quantify HSPCs on functional level, the numbers of colony-forming unit cells (CFU-C) were assessed. Briefly, 2.5 × 10^4^ cells per replicate were resuspended in Iscove’s Modified Dulbecco’s Medium (IMDM) (Thermo Fisher Scientific) with 2% fetal bovine serum (FBS) (Sigma-Aldrich Chemie GmbH, Taufkirchen, Germany) and plated in duplicates into methylcellulose-based medium (MethoCult H4434 Classic; Stemcell Technologies SARL, Saint Égrève, France) in 35 mm cell culture dishes. Cultures were maintained at 37 °C in humidified atmosphere with 5% CO_2_ for 10–16 days. Colonies were counted and evaluated by inverted microscopy with tenfold magnification, thereby differentiating burst-forming unit-erythroid (BFU-E), colony-forming unit-macrophage (CFU-M), colony-forming unit-granulocyte, macrophage (CFU-GM), and colony-forming unit-granulocyte, erythroid, macrophage, megakaryocyte (CFU-GEMM). Using the known sample volume, the input cell count and the TNC, the number of colonies counted was converted to CFU-C per milliliter of processed BM applying the rule of three.

### Colony-forming unit fibroblast assay

To determine the MSC progenitor content, the numbers of colony-forming unit fibroblasts (CFU-F) were assessed. In detail, 7.5 × 10^5^ cells per replicate were resuspended in standard cell culture medium, composed of Alpha MEM (Lonza), 10% human platelet lysate (hPL) (manufactured in-house), 2 IU/mL heparin (Ratiopharm, Ulm, Germany), and 1% penicillin–streptomycin (Thermo Fisher Scientific) and seeded in duplicates in 2 wells of a 6-well plate. Cultures were maintained at 37 °C in humidified atmosphere with 5% CO_2_ for 7–8 days. CFU-F were stained with 0.5% crystal violet solution (Sigma-Aldrich) and counted.

Using the known sample volume, the input cell count and the TNC, the number of colonies counted was converted to CFU-F per milliliter of processed BM applying the rule of three.

### Projection models for TNC and CD34pos target dose and MSC dose manufacturing

The required BM volume for a given HPC-M product was projected using the formulae

$$2 \times {10}^{8}\;\mathrm{TNCs }\times \mathrm{recipient\;BW\;}(\mathrm{kg}) \div \mathrm{TNCs}/\mathrm{mL\;processed\;BM}$$

and

$$4 \times {10}^{6}\;\mathrm{CD}34\mathrm{pos }\times \mathrm{\;recipient\;BW\;}(\mathrm{kg}) \div \mathrm{\;CD}34\mathrm{pos}/\mathrm{mL\;processed\;BM}$$

The projected required BM volume was then used to calculate the respective expected Hb loss.

The number of MSC doses at passage (P) 3 that can be manufactured from a given BM volume was calculated as follows.

Based on the flow cytometry data presented here, up to 0.1% of the TNCs are MSC precursors. Thus, multiplying 0.1% of TNC per milliliter processed BM by the given BM volume gives the maximum number of MSCs that can be obtained at the end of P0 (= N_0_).

For the following passages, the projected MSC yield was calculated according to the formula *N* = *N*_*0*_ × *2*^*n*^, where *N* is the projected MSC yield at end of the respective passage, N_0_ is the projected MSC yield at P0, and *n* are the cumulative population doublings (cPDs) of the respective passage, i.e., number of cumulative doublings occurring in this passage. For the calculation, we used representative cPDs for MSCs as reported previously [19].

### Statistics

Quantitative data are presented as means ± standard error of the mean (SEM) unless stated otherwise and were, following testing for normal distribution, compared with two-tailed *t*-tests (Fig. 2), Wilcoxon matched pairs tests (Figs. 3, 4, 5, 6, and 7B, D, Supplementary Figs. 4-6A, 7–9), analyses of variance (ANOVA) (Fig. 1, Supplementary Figs. 2, 6B/C), and post hoc tests as specified in the figures using GraphPad Prism (La Jolla, CA, USA). Exact *p*-values are reported in the figures, and *p* < 0.05 was considered as statistically significant.

**Fig. 1:**
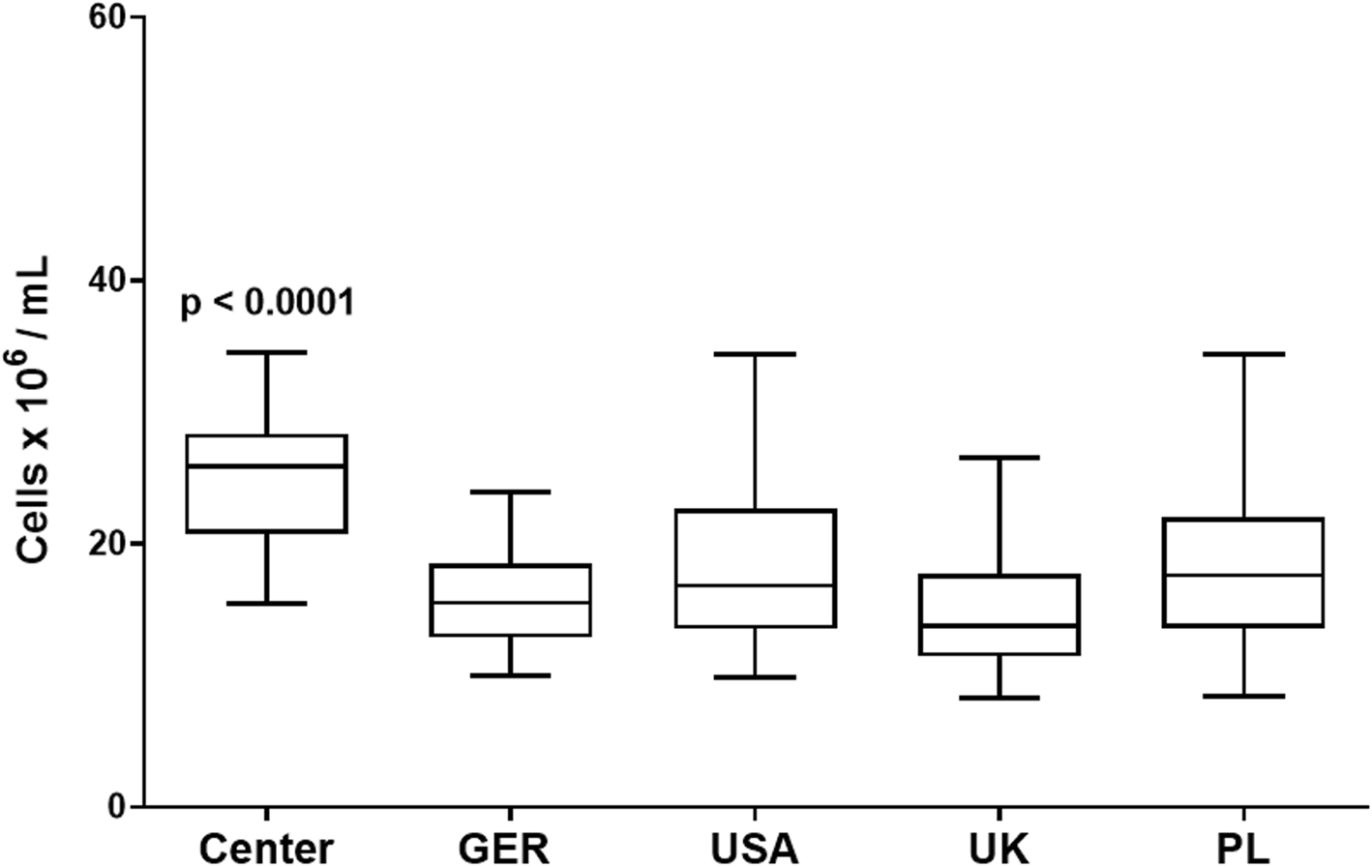
Total nucleated cell concentrations in HPC-M products collected in our center and in other international centers. One-way ANOVA (Kruskal–Wallis test) followed by Dunn’s multiple comparisons test; horizontal lines: means; error bars: 5–95 percentile; *N* = 81 for our center (center), *N* = 829 for GER (Germany without our center), *N* = 96 for USA, *N* = 33 for UK, *N* = 209 for PL

For each sampling time (t1, t2, t3), we calculated the quotients of concentration of a cell type in small to large volume aspirates. With these ratios, we performed non-parametric Spearman correlation analysis (GraphPad Prism) for TNCs versus cell population of interest to assess whether the enrichment of various cell types in small volume aspirates is solely due to the increased content of TNCs.

To visualize the relationship of a cell population’s ratio (small/large) to TNC ratio (small/large), we performed linear regression (GraphPad Prism) and showed a perfect fit line (TNCs) for comparison, when TNCs alone would explain the enrichment of different cell populations in small volume aspirates.

The cartoon (Supplementary Fig. 3) was created with BioRender.

## Results

### Total nucleated cell yields in HPC-M products vary substantially between collection centers suggesting an impact of bone marrow aspiration techniques on HPC-M quality

When we compared HPC-M products collected at our center to those collected in other centers worldwide for DKMS donors in 2019, we found that the TNC contents varied substantially. Specifically, the HPC-M products that were collected in our center had higher TNC concentrations (average of 25.01 × 10^6^/mL) and higher TNC counts (average of 22.89 × 10^9^ TNCs per product) when compared to other international centers (Fig. 1, Additional file 1: Fig. S2A). Such, we met, or even exceeded, the requested TNC numbers in more cases than other centers (Additional file 1: Fig. S2B). For several years, we have implemented a standard technique for BM collections allowing a maximal volume of 5 mL for each individual BM aspirate, whereas other centers may manufacture the HPC-M products with larger volume BM aspirates. This prompted us to test our hypothesis that small volume BM aspirates are higher concentrated for nucleated cells, whereas the larger BM aspirate volumes contain more RBCs per milliliter, thus collecting fewer nucleated cells per volume unit (Additional file 1: Fig. S3).

### Bone marrow collected with small volume aspirates contains more total nucleated cells, immune cells, and platelets

**Fig. 2:**
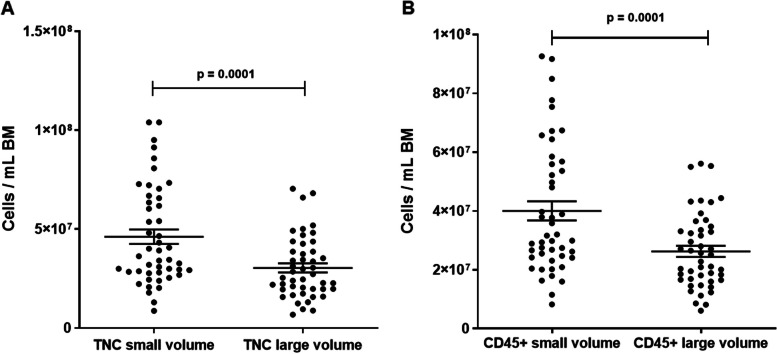
Total nucleated cells and CD45pos cells per mL BM in small volume aspirates vs. large volume aspirates. **A** TNC concentrations, paired *t*-test; dots: individual samples; horizontal lines: means; error bars: SEM; 20 donors, 1–3 time points, 2 volumes each; small volume samples: *N* = 47; large volume samples: *N* = 46. **B** CD45pos concentrations. Paired *t*-test; dots: individual samples; horizontal lines: means; error bars: SEM; 20 donors, 1–3 time points, 2 volumes each; small volume samples: *N* = 46; large volume samples: *N* = 46

**Fig. 3:**
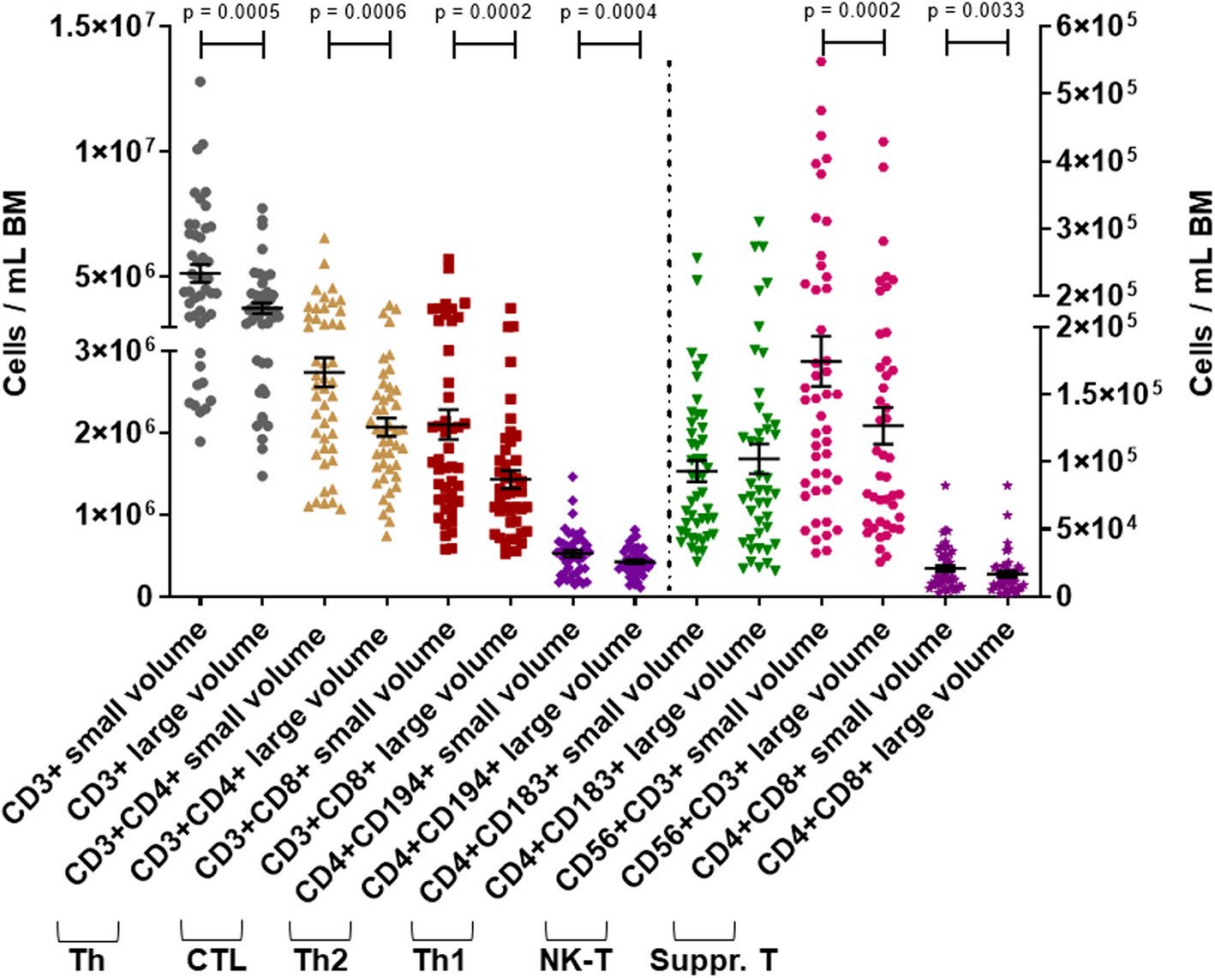
T cells per mL BM in small volume aspirates vs. large volume aspirates. Wilcoxon matched pairs test; dots: individual samples; horizontal lines: means; error bars: SEM; right *Y*-axis refers to Th1 cells, NK-T cells, and suppressor T cells; 20 donors, 1–3 time points, 2 volumes each; small volume samples: *N* = 46; large volume samples: *N* = 44

First, we analyzed the concentrations of TNCs per mL BM in small volume aspirates and large volume aspirates. We found that each milliliter of small volume aspirates contained significantly more TNCs than large volume aspirates (average of 4.62 × 10^7^ [small] vs. 3.04 × 10^7^ [large]; *p* = 0.0001) (Fig. 2A). Accordingly, the concentration of CD45 + cells was higher in the small volume aspirates (average of 4.0 × 10^7^/mL [small] vs. 2.62 × 10^7^/mL [large]; *p* = 0.0001) (Fig. 2B). Next, we aimed to investigate the immune cell composition of the BM and if we would find differences between the small and large volume aspirates. Therefore, we analyzed the CD45 + population for immune cell subsets and identified neutrophils as most frequent immune cell types followed by T cells in small and large volume aspirates. Both neutrophils (average of 2.64 × 10^7^/mL [small] vs. 1.74 × 10^7^/mL [large]; *p* = 0.0003) and T cells (average of 5.14 × 10^6^/mL [small] vs. 3.75 × 10^6^/mL [large]; *p* = 0.0005) were higher concentrated in small volume aspirates (Additional file 1: Fig. S4A; Fig. 3). Further characterizing the immune cell subsets by multicolor flow cytometry, we found that B cells (average of 2.67 × 10^6^/mL [small] vs. 1.59 × 10^6^/mL [large]; *p* = 0.0003), NK cells (average of 1.68 × 10^6^/mL [small] vs. 0.97 × 10^6^/mL [large]; *p* = 0.0001), monocytes/macrophages (average of 1.44 × 10^6^/mL [small] vs. 0.82 × 10^6^/mL [large]; *p* = 0.0002), cytotoxic T cells (CTLs) (average of 2.1 × 10^6^/mL [small] vs. 1.43 × 10^6^/mL [large]; *p* = 0.0002), NK-T cells (average of 1.75 × 10^5^/mL [small] vs. 1.27 × 10^5^/mL [large]; *p* = 0.0002), and suppressor T cells (average of 2.08 × 10^4^/mL [small] vs. 1.65 × 10^4^/mL [large]; *p* = 0.0033) were significantly higher concentrated in small volume aspirates compared to large volume aspirates (Additional file 1: Fig. S5; Fig. 3). We also observed higher concentrations of T Helper (Th) cells in small volume aspirates (average of 2.73 × 10^6^/mL [small] vs. 2.07 × 10^6^/mL [large]; *p* = 0.0006). Interestingly, the concentration effect in the small volume aspirate pertained only to Th_2_ cells (average of 5.24 × 10^5^/mL [small] vs. 4.21 × 10^5^/mL [large]; *p* = 0.0004), whereas Th_1_ cells trended higher in large volume aspirates (average of 0.93 × 10^5^/mL [small] vs. 1.02 × 10^5^/mL [large]; *p* = 0.4024 (Fig. 3). In addition, the platelet concentrations were higher in the small volume aspirates compared to the large volume aspirates (average of 1.53 × 10^8^/mL [small] vs. 1.07 × 10^8^/mL [large]; *p* = 0.0003) (Additional file 1: Fig. S4B).

### Small volume bone marrow aspirates concentrate hematopoietic stem/progenitor cells

**Fig. 4:**
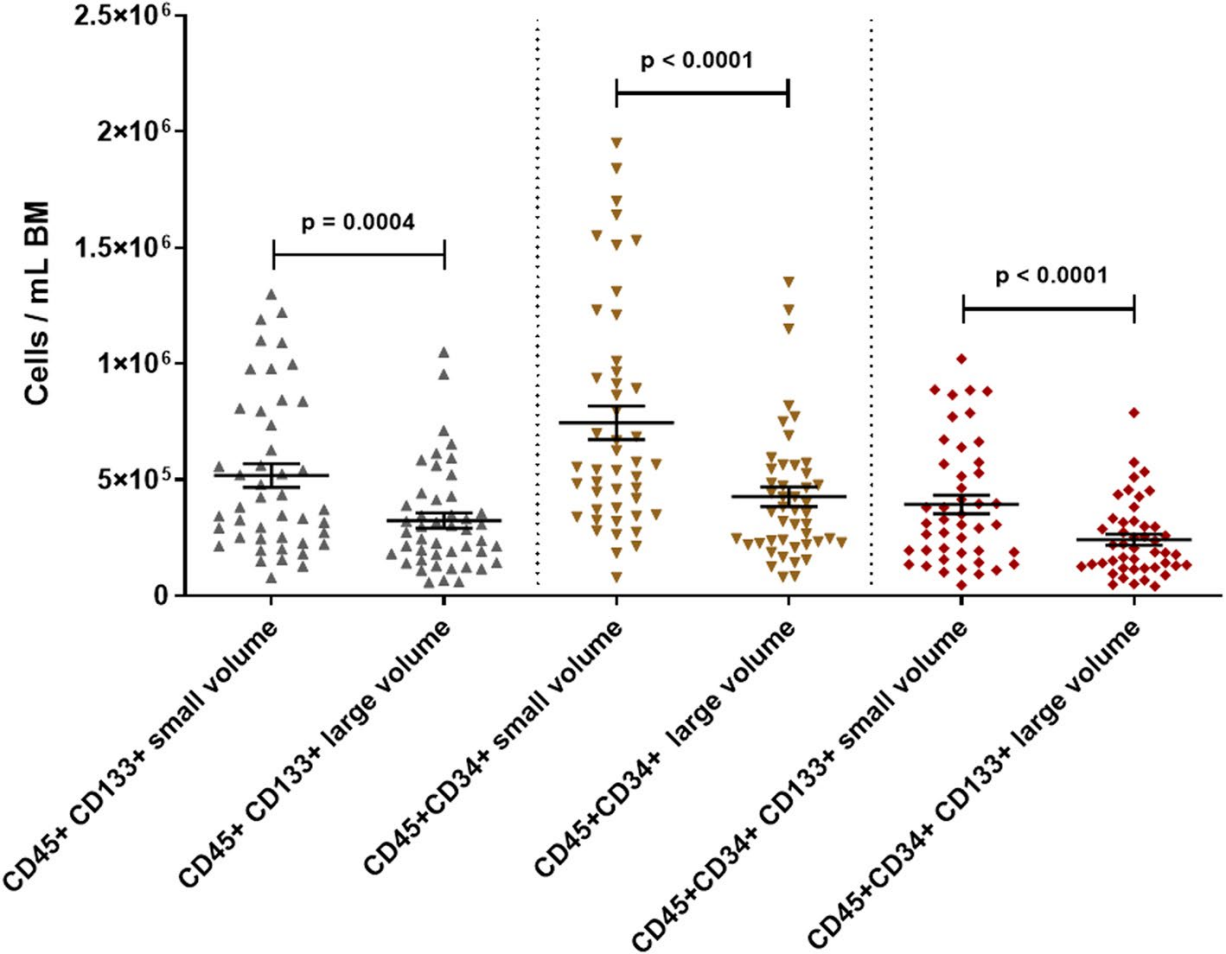
Hematopoietic stem/progenitor cells per mL BM in small volume aspirates vs. large volume aspirates. Wilcoxon matched pairs test; dots: individual samples; horizontal lines: means; error bars: SEM; 20 donors, 1–3 time points, 2 volumes each; small volume samples: *N* = 45–46; large volume samples: *N* = 46

We showed that, compared to large BM volume aspirates, small volume aspirates concentrate TNCs, most immune cell types and platelets. Therefore, we tested if HSPCs would also follow this principle. Identified by multicolor flow cytometry, we found substantially more CD34pos (average of 7.45 × 10^5^ [small] vs. 4.26 × 10^5^ [large]; *p* < 0.0001), CD133pos (average of 5.18 × 10^5^ [small] vs. 3.24 × 10^5^ [large]; *p* = 0.0004), and CD34posCD133pos (average of 3.93 × 10^5^ [small] vs. 2.41 × 10^5^ [large]; *p* < 0.0001) cells per milliliter in small volume aspirates (Fig. 4).

**Fig. 5:**
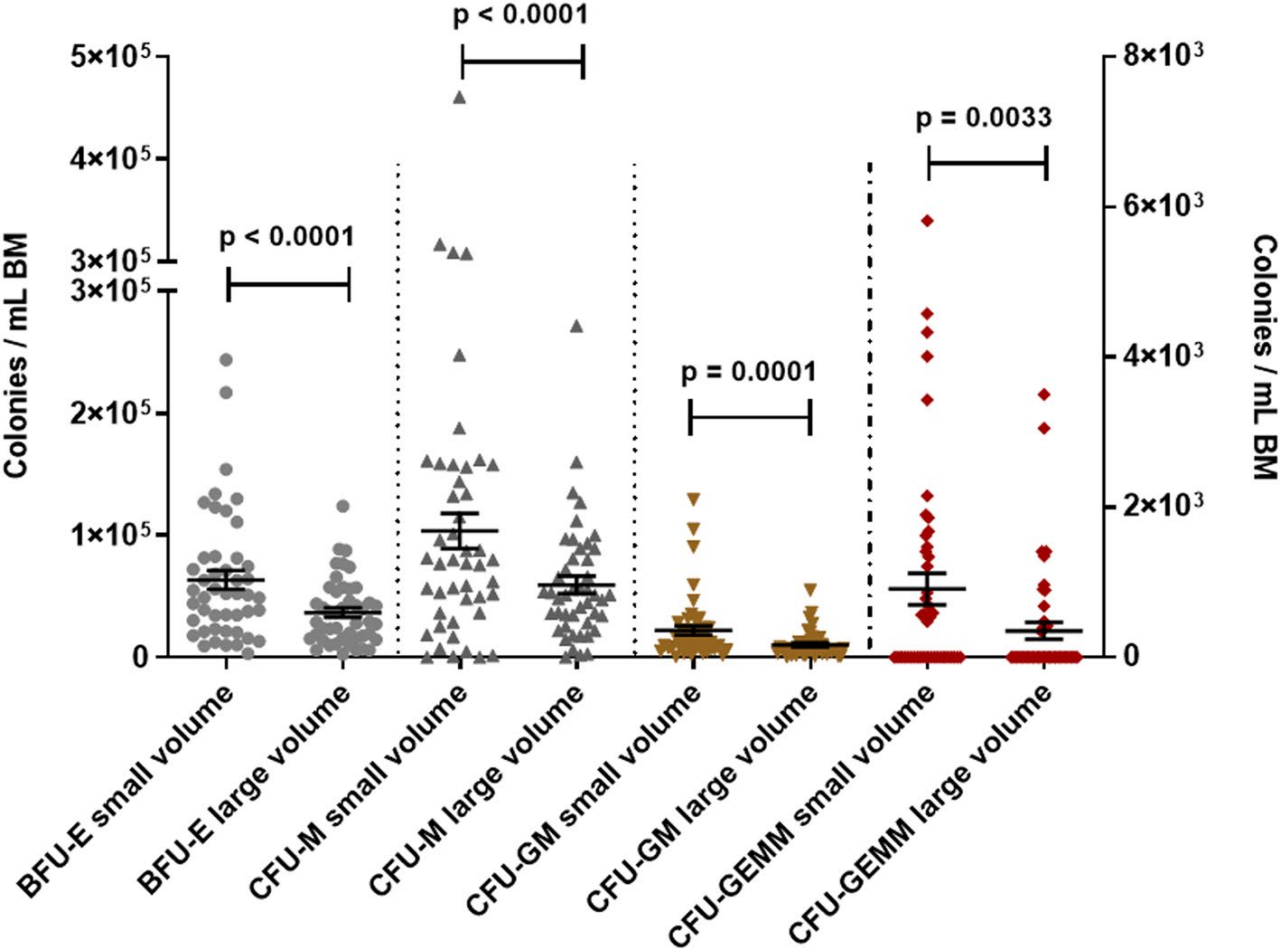
Hematopoietic stem/progenitor cell colonies per mL BM in small volume aspirates vs. large volume aspirates. Wilcoxon matched pairs test; dots: individual samples; horizontal lines: means; error bars: SEM; right *Y*-axis refers to CFU-GEMM; 19 donors, 1–3 time points, 2 volumes each; small volume samples: *N* = 46; large volume samples: *N* = 46

To confirm the stem/progenitor character of the cells, we additionally quantified the hematopoietic colony forming HSPC clones. In line with the multicolor flow cytometry results, small volume BM aspirates had higher concentrations of BFU-E (average of 6.34 × 10^4^/mL [small] vs. 3.66 × 10^4^/mL [large]; *p* < 0.0001), CFU-M (average of 1.04 × 10^5^/mL [small] vs. 0.59 × 10^5^/mL [large]; *p* < 0.0001), CFU-GM (average of 2.21 × 10^4^/mL [small] vs. 1.02 × 10^4^/mL [large]; *p* = 0.0001), and CFU-GEMM (average of 9.11 × 10^2^/mL [small] vs. 3.52 × 10^2^/mL [large]; *p* = 0.0033) (Fig. 5). The concentrations of HSPCs in both small volume and large volume BM aspirates decreased over time suggesting a kinetic of HSPC harvest during the individual collection process. Yet, the reduction of HSPC concentration was significant only for large volume aspirates, and the small volume aspirates contained still significantly more HSPCs per milliliter at each time point during the individual BM collections (Additional file 1: Fig. S6).

### The concentration effect of small volume bone marrow aspirates applies also to non-hematopoietic progenitor cells

**Fig. 6:**
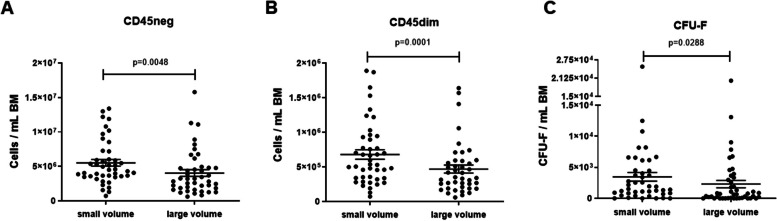
Mesenchymal stromal cells and mesenchymal stromal cell precursors per mL BM in small volume aspirates vs. large volume aspirates. **A** CD45 negative cells. **B** CD45 dim cells. **C** MSC precursors (CFU-F). Wilcoxon matched pairs test; dots: individual samples; horizontal lines: means; error bars: SEM; 19 donors, 2 volumes each; small volume samples: *N* = 43–44; large volume samples: *N* = 43–45

The majority of the tested cell types were higher concentrated in the small volume aspirate; however, this was not the case for all cell types. Thus, we assessed, as a next step, the concentration effect of small volume aspirates for additional, non-hematopoietic progenitor cells types that have been increasingly used for cell therapies manufacture, i.e., MSCs and EPCs. In the human BM, MSC populations and their precursors can be found in the CD45neg and CD45dim fractions [20, 21]. Both were higher concentrated in the small volume aspirates (CD45neg: average of 5.52 × 10^6^/mL [small] vs. 4.04 × 10^6^/mL [large]; *p* = 0.0048; CD45dim: average of 6.79 × 10^5^/mL [small] vs. 4.69 × 10^5^/mL [large]; *p* = 0.0001) (Fig. 6A, B). As MSCs feature substantial heterogeneity, we quantified the MSC subpopulations with multicolor flow cytometry and found for both CD45neg and CD45dim cells the CD146posCD29pos, CD146posCD119pos, and CD271posCD90pos as most prevalent subpopulation phenotypes. We detected all MSC subpopulations at higher concentrations in the small volume aspirates (Additional file 1: Fig. S7 + 8). Quantifying the MSC progenitors by CFU-F, we confirmed the concentration effect of the small volume aspirates for MSCs (average of 3.47 × 10^3^/mL [small] vs. 2.30 × 10^3^/mL [large]; *p* = 0.0288) (Fig. 6C). Regarding endothelial cell precursors, we found more CD31posCD45negCD14negCD133negCD34pos ECFCs (average of 1.41 × 10^5^ [small] vs. 1.05 × 10^5^/mL [large]; *p* < 0.0001) per milliliter in small volume aspirates compared to large volume aspirates (Additional file 1: Fig. S9).

### The hemoglobin and red blood cell concentrations are higher in large volume aspirates than in small volume aspirates

**Fig. 7:**
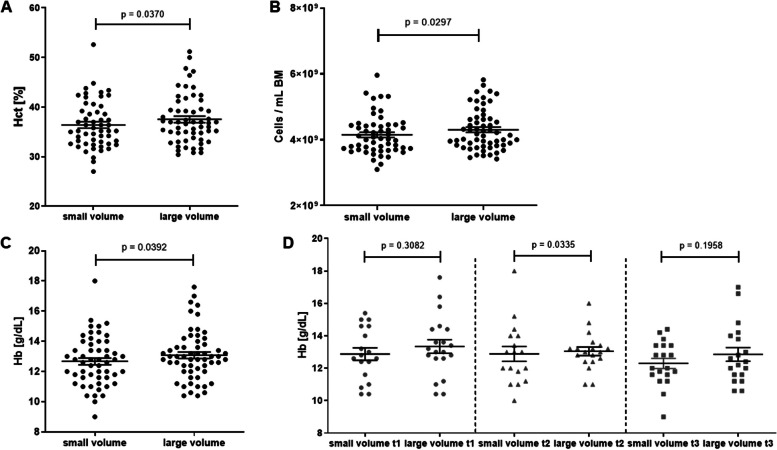
Hemoglobin, hematocrit, and RBCs in small volume aspirates vs. large volume aspirates. **A** Cumulative Hct over all time points. **B** Cumulative RBC counts over all time points. **C** Cumulative Hb over all time points. **D** Hb at different time points. Wilcoxon matched pairs test, except for cumulative Hb (paired *t*-test); dots: individual samples; horizontal lines: means; error bars: SEM; 20 donors, 2 volumes each; small volume samples: *N* = 54; large volume samples: *N* = 58

Next, we asked, if the lower concentration of the nucleated cells that we observed in the large volume aspirates could come with a higher plasma fraction or more RBCs that would compensatory add up to the aspirate volume. Therefore, we analyzed the Hct as well as the Hb and RBC concentrations. We recorded higher Hct values (average of 37.52% [large] vs. 36.40% [small]; *p* = 0.0370) in the large volume aspirates (Fig. 7A), which excluded a substantial plasma dilution in these samples, but pointed toward RBCs as the main source contributing to the lower TNC concentrations in the large volume aspirates. Indeed, we found more RBCs per milliliter (average of 4.307 × 10^9^ [large] vs. 4.150 × 10^9^ [small]; *p* = 0.0297) (Fig. 7B) and more Hb per deciliter (average of 13.08 g [large] vs. 12.67 g [small]; *p* = 0.0392) in the large volume aspirates than in small volume aspirates (Fig. 7C), where the latter did not change during the time course of the BM collection process (Fig. 7D).

Manufacture of cell therapeutics with small volume bone marrow aspirates substantially reduces the required bone marrow volume, increases manufacture efficiency, and minimizes hemoglobin loss for donors.

**Fig. 8:**
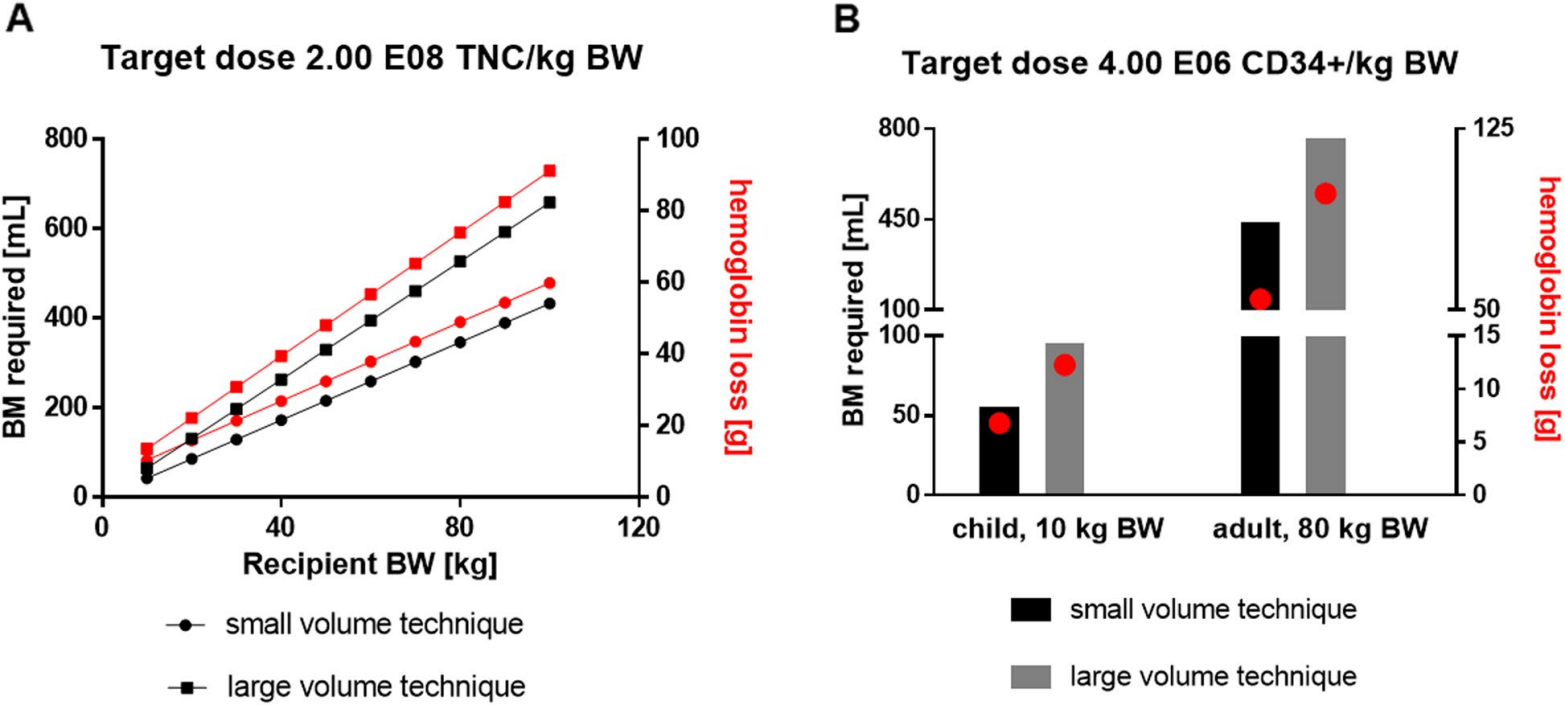
Modeling required BM volume and expected hemoglobin loss for clinical target doses collected with small volume aspirates vs. large volume aspirates. **A** Target dose 2 × 10^8^ TNC/kg BW. Red dots indicate Hb loss in g. **B** Target dose 4 × 10^6^ CD34pos/kg BW. Red dots indicate Hb loss in g

We observed that small volume aspirates concentrate nucleated cells, including HSPCs and MSCs, and contain less Hb per volume unit. In order to assess the clinical relevance of our findings, we projected the required BM volume and expected Hb loss for various TNC and CD34pos HPC-M target doses for both small and large volume aspirates. We found that small volume BM aspirates save up to 42% BM volume and 44% Hb for HPC-M donors with an increasing saving effect at higher patient’s body weight (Fig. 8). We also tested if small volume aspirates would be favorable for BM-MSC therapeutics production. Projecting MSC doses that could be manufactured from a given BM volume, we found that the production efficiency was more than 150% higher, for both pediatric and adult patients, with small volume aspirates compared to large volume aspirates (Table 2).

**Table 2:** Comparison of MSC doses that can be manufactured with small volume aspirates and large volume aspirates

**Number of MSC doses that can be manufactured from a given BM volume**	**child @ 10 kg BW** **target dose 1** × **10**^**6**^** MSCs/kg BW**		**child @ 10 kg BW** **target dose 2** × **10**^**6**^** MSCs/kg BW**	
BM volume (mL)	Small volume aspirate	Large volume aspirate	Small volume aspirate	Large volume aspirate
50	294	193	147	97
100	587	386	294	193
500	2937	1931	1468	966
	**% dose production compared to large volume**		**% dose production compared to large volume**	
	**152.04**		**152.04**	
**Number of MSC doses that can be manufactured from a given BM volume**	**adult @ 80 kg BW** **target dose 1** × **10**^**6**^** MSCs/kg BW**		**adult @ 80 kg BW** **target dose 2** × **10**^**6**^** MSCs/kg BW**	
BM volume (mL)	Small volume aspirate	Large volume aspirate	Small volume aspirate	Large volume aspirate
50	37	24	18	12
100	73	48	37	24
500	367	241	184	121
	**% dose production compared to large volume**		**% dose production compared to large volume**	
	**152.04**		**152.04**	

*BW* Body weight

## Discussion

For decades, substantial variabilities in TNC counts have been observed in HPC-M products collected worldwide. With individual centers reporting a multi-decade decline in TNC counts [15], this issue has become a growing concern in the field of BM transplantation, especially when the requested TNC doses are not met. This has initiated a discussion about the underlying cause of this problem.

Benchmarking our BM collection center to others in Europe and the USA, we showed that non-US BM collection cell concentrations and counts trended below respective values from US centers, but we also found that the HPC-M products that we collected had not only the highest TNC concentrations but also met most of the TNC requests (80%) from the transplantation centers.

As the TNC counts of the HPC-M products were performed at the collection centers before shipping, we can rule out potential impact of post collection processing or shipping variables (e.g., travel distance or time) on the TNC data presented in our study. Such, mainly collection and/or donor related factors could have influenced the TNC counts. Regarding the latter, we have included for each collection center HPC-M products from a substantial number of donors, which met internationally consented eligibility and acceptance criteria for BM donation. This leaves the BM collection technique as the variable with the most significant influence on the TNC counts, and such on the HPC-M quality.

Our comparative analysis of small versus large volume BM aspirates show that the small volume aspiration technique, which we have implemented for HPC-M manufacture in our center for years, has a higher efficacy for collecting TNCs and HSPCs. Particularly acknowledging that BM aspiration is a technique, which relies on manual skills, it is important for studies investigating BM collection to eliminate, or at least minimize, operator effects that could affect the endpoints. Therefore, different collection teams performed the BM sampling for our study, with small and large volume samples being taken randomly by different persons. In addition, we analyzed multiple aspirates from twenty allogeneic donors sampled at three time points per donation. Together, these measures have sufficiently minimized possible operator effects and enabled robust statistical analyses.

It is known that higher TNC and CD34pos cell doses correlate with faster neutrophil engraftment after HSCT [22]. However, the quality of BM collection is highly variable, not least due to technical variabilities. Although it appears that the CD34pos cell count in the HPC-M product is linked to the collected BM volume [23], the concrete impact of BM collection techniques on HPC-M quality has not been clarified and, therefore, BM collection is not standardized to date. Specifically, the question if multiple small volume BM aspirations or fewer large volume aspirations would be preferable has been under debate. Yet, so far, surprisingly, few studies have addressed this relevant question with mixed results. One study compared the composition of autologous and allogeneic HPC-M products collected from adults and children with either very low volume (2 mL) or very high volume (100 mL) aspirates [24]. In contrast to our study, they found no differences for mononuclear cells, T cells, and CFU content. However, they detected more CD34pos cells in the HPC-M products manufactured with small volume aspirates, which goes in line with our observations. Another study, similarly designed as the aforementioned study, yet comparing HPC-M collected with 2 mL versus 20 mL aspirates, reported that small volume aspirations yielded higher concentrations of nucleated cells, more CFU-GM, and lower T cell concentrations [25]. Interestingly, they concluded that small volume aspirations minimize the dilution with PB, echoing in this aspect another study that was conducted before [26]. A recent study reported a negative correlation of TNC concentrations in BM samples to the BM harvest volume and to the percentage of donor volume harvested [27]. This supports our herein presented observations, particularly as in this study only large volume (10 mL) aspirations were used for BM collection, which also highlights the advantage of small volume aspirates.

In contrast to TNCs, HSPCs, and other cell types, we noted a higher Hb and RBC content per milliliter in the large volume BM samples. We wondered if the main reason behind this observation would be an increasing influx of PB into the BM aspirate during the collection process as suggested by the above-mentioned previous reports [25, 26]. However, the fact that we observed higher Hb and RBC values in the large volume samples not only at the start but at all time points and that small volume aspirates contained a higher concentration of platelets, which together with RBCs are present in PB, speak against this hypothesis. This conclusion is supported by another study that aimed to test the hypothesis that the CD34pos cell concentration in BM aspirates would decrease during the BM collection procedure due to increasing PB contamination in the aspirates. Specifically, counting CD34pos cells during BM collection at 200-mL intervals, they found no significant variations between the time points [23], which also is in line with our observations. Moreover, the higher Hct values that we recorded in the large volume aspirates excluded a substantial plasma dilution in these samples and suggested RBCs collected from the drilled BM channel, but not the following influx of PB into the channel, as the main factor contributing to the lower TNC concentrations in the large volume aspirates. However, it is clear that the withdrawal of larger BM volumes with a substantial number of RBCs eventually results in greater Hb loss for the donor.

Moreover, the relationship of differing BM aspirate volumes and their therapeutic cellular composition, such as HSPCs, MSCs, EPCs, or immune cell subsets, has remained unclear to date [24–26, 28]. To better understand the physiology underlying the higher concentration efficacy for certain cell types in small volume BM aspirates, we further modeled the collection kinetics of the cell types within the TNC fraction. Therefore, we calculated the concentration ratios of small versus large volume samples for TNCs and compared them to the ratios of the other cell types. For validation, we included CD45pos cells, which, as expected, had identical concentration ratios as TNCs (Additional file 1: Fig. S10). However, some cell types featured substantially different concentration ratios compared to TNCs as shown by the position of their regression lines in relation to the TNC regression line. Specifically, a position of the regression line above the TNC line indicates a higher cell concentration compared to TNCs in the small volume aspirates. This is true for hematopoietic and non-hematopoietic progenitor cells, whereas the regression lines of the T cell subsets are below the TNC line (Additional file 1: Fig. S10-12).

Such, our observations allow us to describe the kinetics of the BM aspiration in detail. At collection start, the cells with lower specific gravity, i.e., platelets and nucleated cells [29], are aspirated. Our model suggests that within these the progenitors are being collected first, followed by more mature cell types such as T cell subsets. Therefore, the small volume aspirates contain higher concentrations of these cell types. With increasing collection volume over time, and fewer cells with lower specific gravity, i.e., platelets, TNCs, HSPCs, MSCs, being available at this location of the needle, more cells with higher specific gravity, i.e., RBCs [29], are taken from the drilled BM channel, hereby adding up to the final volume of the large aspirates (Additional file 1: Fig. S3).

In the past decades, a variety of different needle types has been used for BM aspiration [30, 31]. The Jamshidi needle, introduced in 1971, allows tissue to freely enter the lumen and, therefore, minimizes tissue damage. This device is most widely applied for BM aspiration [32] and was also used in our study. Fenestrated needles that employ multiple small volume draws (1 mL) from a single puncture by promoting lateral flow from multiple sides yielded higher TNC, CD34pos, and CFU-F counts when compared to traditional needle aspirate [33]. Fenestrated needles may be an additional practical application of our proposed specific gravity mechanism due to their small volume draw approach by design. This could further optimize the aspiration of multiple small volumes from different locations in a single puncture channel, hereby reducing the harvesting time of BM that contains more cells of lower specific gravity (TNCs, CD34pos cells) and fewer cells of high specific gravity (RBCs). Further studies are suggested to evaluate this option.

Another cell therapeutic with increasingly promising potential for both immunomodulation and regenerative medicine applications are MSCs, which, for the majority of clinical trials, are obtained from the BM [7, 34, 35]. In the BM aspirates that we analyzed, we found that 0.1% of the TNCs were MSC precursors, which corresponds to the previously reported frequency [36]. Yet, the relatively low MSC numbers that can be harvested from the BM require their ex vivo expansion to manufacture enough MSC doses for clinical trials treating multiple patients [19, 37]. Even when applying advanced manufacturing strategies, such as innovative pooling concepts, to maximize the MSC yield at lowest possible passage [37], the MSC number at the start of the expansion process is critical. We demonstrate that the principle of collecting maximal numbers of cells with minimal BM volume taken from the donor is not only achievable for HSPCs but is also applicable to other nucleated cells such as MSCs. Specifically, with small volume BM aspirates, more MSCs can be collected with the same BM volume and, thus, expanded for clinical dose production, which can be tripled compared to large volume aspirates. In the same way, a lower BM volume needs to be collected to isolate the required MSC number for up-scaling production. To collect the equal BM volume, fewer large volume aspirates are needed, which can be more quickly obtained than small volume aspirates [28]. We show that, due to higher cell concentration, the total BM that eventually needs to be collected is lower with small volume aspirates compared to large volume aspirates but could increase, to a certain extent, total collection time and anesthesia duration for the donor, particularly when BM is harvested for patients with higher body weight. This should also be considered when assessing the safety of the BM collection technique. A specific clinical problem is the burden for pediatric donors, who have a high risk of significant Hb loss due to BM collection [38]. Specifically, more than 50% of pediatric BM donors require RBC transfusions [39]. It can be speculated that, in some cases, even well-matched pediatric donors may be excluded from donation as their low body weight does not allow to collect higher BM volumes. Therefore, together with our previously reported BM donor blood management concept [40], consequently collecting small volume BM aspirates contributes to donor safety and well-being.

As MSCs feature substantial heterogeneity in vivo as well as in vitro [41–44], we analyzed the frequencies of MSC subsets in the BM aspirates. The CD146(MCAM)pos subset, defining bone, cartilage, and stromal progenitors in vivo [45], features superior migration capacity [46], a trait being linked to MSC therapeutic efficacy [47], and increased production of anti-inflammatory proteins resulting in enhanced immunomodulation potential [48]. Compared to unselected MSCs, the CD271positively selected subpopulation was shown to stronger suppress immune cell activity, featured high migration and proliferation capacity, and may contain more osteoprogenitors as supported by gene expression and functional analyses [21, 49–52]. We identified in both, the CD45neg and the CD45dim fractions of the BM aspirates, CD146posCD29pos, CD146posCD119pos, and CD271posCD90pos cells as most frequent MSC phenotypes, with all being higher concentrated in the small volume aspirates, which may add another qualitative superiority of this technique.

Yet, not all cell types were significantly higher concentrated in the small volume aspirates. Regarding endothelial progenitor cell subsets, we found, in contrast to other nucleated cells including neutrophils, that the small volume technique concentrated only ECFCs, but not CACs or EPCs. Still, with an average of 1 × 10^5^ cells, more ECFCs can be harvested with both aspiration techniques in 1 mL BM compared to approximately 180 ECFCs in a complete apheresis unit from steady state PB [53].

It was shown previously that engraftment and recipient long-term outcomes depend on the transplanted CD34pos cell dose [22, 54], and we herein report that the target TNC, CD34pos cell dose can be reached with less BM collected with small volume aspirations. Such, we extrapolate that the clinical outcomes of patients who received HPC-M products collected with small volume aspirates would not be inferior to patients who received products of the same TNC, CD34pos cell dose collected with large volume aspirates. To confirm our findings in the clinic, we propose as next step studies specifically comparing the outcomes of patients who received either small volume aspirate HPC-M or large volume HPC-M of identical TNC, CD34pos cell doses.

There are some limitations of our study to be considered. While our investigation focused on the impact of BM collection technique on the yield and composition of harvested cells, the correlation of these findings with clinical outcomes such as engraftment, GvHD and overall patient survival remains to be determined.

Furthermore, in our study, we used a single type of BM aspiration needle, albeit one of the most commonly employed varieties. As mentioned above, variations in needle type could potentially introduce nuances in the collection process and influence cell yields and compositions. Exploring the effects of different needle types could provide a more comprehensive understanding of how collection techniques impact cell populations.

Another limitation pertains to the demographic profile of our donor cohort, which was comprised exclusively of adult individuals. The impact of BM volume aspiration on pediatric donors might differ due to their anatomical and physiological characteristics.

It is also important to acknowledge that the prospective part of our study was conducted at a single center. While our findings demonstrate the efficacy of small volume aspiration in our specific setting, the generalizability of these results to other centers with possibly varying conditions requires further validation through multi-center studies.

Lastly, our study design, sampling both small and large volume aspirates from the same donor to mitigate donor variability, limited our ability to assess potential differences in the duration of anesthesia required for each technique. Future studies employing a larger donor cohort and comparing donors with small volume aspirates to donors with large volume aspirates could offer insights into how the choice of collection technique might influence anesthesia-related factors.

## Conclusions

Our investigation offers valuable insights into the benefits of small volume BM aspiration covering the relevant cell types for hemato-oncologic, immunomodulatory, and regenerative clinical therapeutics which can be manufactured from BM. Future multi-center studies could contribute to a more holistic understanding of the implications and benefits of adopting small volume aspiration as a standard approach for BM collection.

### Supplementary Information

**Additional file 1: Fig. S1. **Sampling overview. For each BM collection three small volume BM aspirates (Punctions A, C and E) as well as three large volume BM aspirates (Punctions B, D and F) were sampled at three time points. To standardize sampling and to exclude possible impact of the needle position in the punction canal each sample was taken from separate punctions as first aspirate after reaching the BM cavity. *N* = 20 donors. **Fig. S2.** Benchmarking HPC-M collection centers worldwide. A. TNC absolute numbers in HPC-M collected in our center (Center) and in other international collection centers. B. Ratio collected to requested TNC numbers in HPC-M collected in our center and in other international collection centers. One-way ANOVA (Kruskall-Wallis test) followed by Dunn’s multiple comparisons test; error bars: 5 - 95 percentile; *N* = 81 for our center (Center), *N* = 829 for GER (Germany without our center), *N*= 96 for USA, *N* = 33 for UK, *N* = 209 for PL.  **Fig. S3.** Hypothesis. Visualization of the hypothesis that small volume BM aspirates are higher concentrated for progenitor cells compared to large volume BM aspirates that contain more red blood cells thus collecting fewer progenitor cells per milliliter. The main defining factor for the cell types being differentially collected is their specific gravity. **Fig. S4.** Neutrophils and platelets in small volume aspirates vs. large volume aspirates. A. Neutrophils per mL BM in small volume aspirates vs. large volume aspirates. B. Platelets per mL BM in small volume aspirates vs. large volume aspirates. Wilcoxon matched pairs test; dots: individual samples; horizontal lines: means; error bars: SEM; 20 donors, 1-3 time points, 2 volumes each; small volume samples: *N* = 47 (neutrophils), *N* = 57 (platelets); large volume samples: *N* = 46 (neutrophils), *N* = 58 (platelets). **Fig. S5.** Mature CD45pos immune cells in small volume aspirates vs. large volume aspirates. Wilcoxon matched pairs test; dots: individual samples; horizontal lines: means; error bars: SEM; 20 donors, 1-3 time points, 2 volumes each; small volume samples: *N* = 46; large volume samples: *N* = 44. **Fig. S6.** Kinetics of HSPC collection in small volume aspirates vs. large volume aspirates. A. CD34pos HSPCs per mL BM in small volume aspirates vs. large volume aspirates at different time points. Wilcoxon matched pairs test; dots: individual samples; horizontal lines: means; error bars: SEM; t1: small volume samples: *N* = 14 donors, large volume samples: *N* = 15 donors; t2: small volume samples: *N* = 15 donors, large volume samples: *N* = 15 donors; t3: small volume samples: *N*= 17 donors, large volume samples: *N* = 16 donors. B. Time course of CD34pos HSPCs per mL BM in small volume aspirates during BM collection. ANOVA followed by Tukey's multiple comparisons test; dots: means; error bars: SEM; t1: *N* = 14 donors; t2: *N* = 15 donors; t3: *N* = 17 donors. C. Time course of CD34pos HSPCs per mL BM in large volume aspirates during BM collection. ANOVA followed by Tukey's multiple comparisons test; dots: means; error bars: SEM; t1: *N* = 15 donors t2: *N* = 15 donors t3: *N*= 16 donors. **Fig. S7.** CD45neg MSC subsets in small volume aspirates vs. large volume aspirates. Wilcoxon matched pairs test; dots: individual samples; horizontal lines: means; error bars: SEM; 19 donors, 1-3 time points, 2 volumes each; small volume samples: *N* = 44; large volume samples: *N* = 39. **Fig. S8.** CD45dim MSC subsets in small volume aspirates vs. large volume aspirates. Wilcoxon matched pairs test; dots: individual samples; horizontal lines: means; error bars: SEM; 19 donors, 1-3 time points, 2 volumes each; small volume samples: *N* = 44; large volume samples: *N* = 39. **Fig. S9.** Endothelial progenitor cell subsets in small volume aspirates vs. large volume aspirates. Wilcoxon matched pairs test; dots: individual samples; horizontal lines: means; error bars: SEM; all cells were positive for CD31 and negative for CD45 and CD14; right Y-axis refers to ECFCs, CACs and EPCs; 20 donors, 1-3 time points, 2 volumes each; small volume samples: *N* = 47; large volume samples: *N* = 46. **Fig. S10.** Correlation of TNC count ratios to hematopoietic subsets count ratios at different time points. Red lines: TNC count ratios (small volume to large volume aspirates). Black lines: Hematopoietic subsets count ratios (small volume to large volume aspirates). Position of the regression line above the TNC line indicates a higher cell concentration compared to TNCs in the small volume aspirates and vice versa. Individual data points are only shown for hematopoietic subset ratios. Spearman Correlation: *N*= 37-41. **Fig. S11.** Correlation of TNC count ratios to CFU count ratios at different time points. Red lines: TNC count ratios (small volume to large volume aspirates). Black lines: CFU count ratios (small volume to large volume aspirates). Position of the regression line above the TNC line indicates a higher cell concentration compared to TNCs in the small volume aspirates and vice versa. Individual data points are only shown for CFU count ratios. Spearman Correlation: *N* = 33-41. **Fig. S12.** Correlation of TNC count ratios to EPC count ratios at different time points. Red lines: TNC count ratios (small volume to large volume aspirates). Black lines: EPC count ratios (small volume to large volume aspirates). Position of the regression line above the TNC line indicates a higher cell concentration compared to TNCs in the small volume aspirates and vice versa. Individual data points are only shown for EPC count ratios. Spearman Correlation: CACs: CD45negCD14negCD31posCD34posCD133pos. EPCs: CD45negCD14negCD31posCD34posCD133posCD309pos. ECFCs: CD45negCD14negCD31posCD34posCD133neg. *N* = 30-41.

## Data Availability

Due to data protection law individual donor data cannot be disclosed. De-identified processed data that underlie the results reported in this article could be shared upon reasonable request from the corresponding author after signing a data protection agreement and a data access agreement.

## Abbreviations

ANOVA: Analyses of variance
BFU-E: Burst-forming unit-erythroid
BM: Bone marrow
BW: Body weight
CFU-C: Colony-forming unit cells
CFU-F: Colony-forming unit fibroblasts
CFU-GEMM: Colony-forming unit-granulocyte, erythroid, macrophage, megakaryocyte
CFU-GM: Colony-forming unit-granulocyte, macrophage
CFU-M: Colony-forming unit-macrophage
cPDs: Cumulative population doublings
EPCs: Endothelial progenitor cells
FBS: Fetal bovine serum
GvHD: Graft-versus-host disease
Hb: Hemoglobin
HPC-M: Hematopoietic progenitor cell product—bone marrow
hPL: Human platelet lysate
HSCT: Hematopoietic stem cell transplantation
HSPCs: Hematopoietic stem/progenitor cells
MSCs: Mesenchymal stromal cells
P: Passage
PB: Peripheral blood
PBSC: Hematopoietic stem/progenitor cells from peripheral blood
RBCs: Red blood cells
SEM: Standard error of the mean
TNC: Total nucleated cell
