# Supplementary material for: Small volume bone marrow aspirates with high progenitor cell concentrations maximize cell therapy dose manufacture and substantially reduce donor hemoglobin loss
Source: BMC Med. 2023 Sep 19;21:360. doi: 10.1186/s12916-023-03059-3 (PMC10510270; doi:10.1186/s12916-023-03059-3)

Additional file 1: Fig. S1

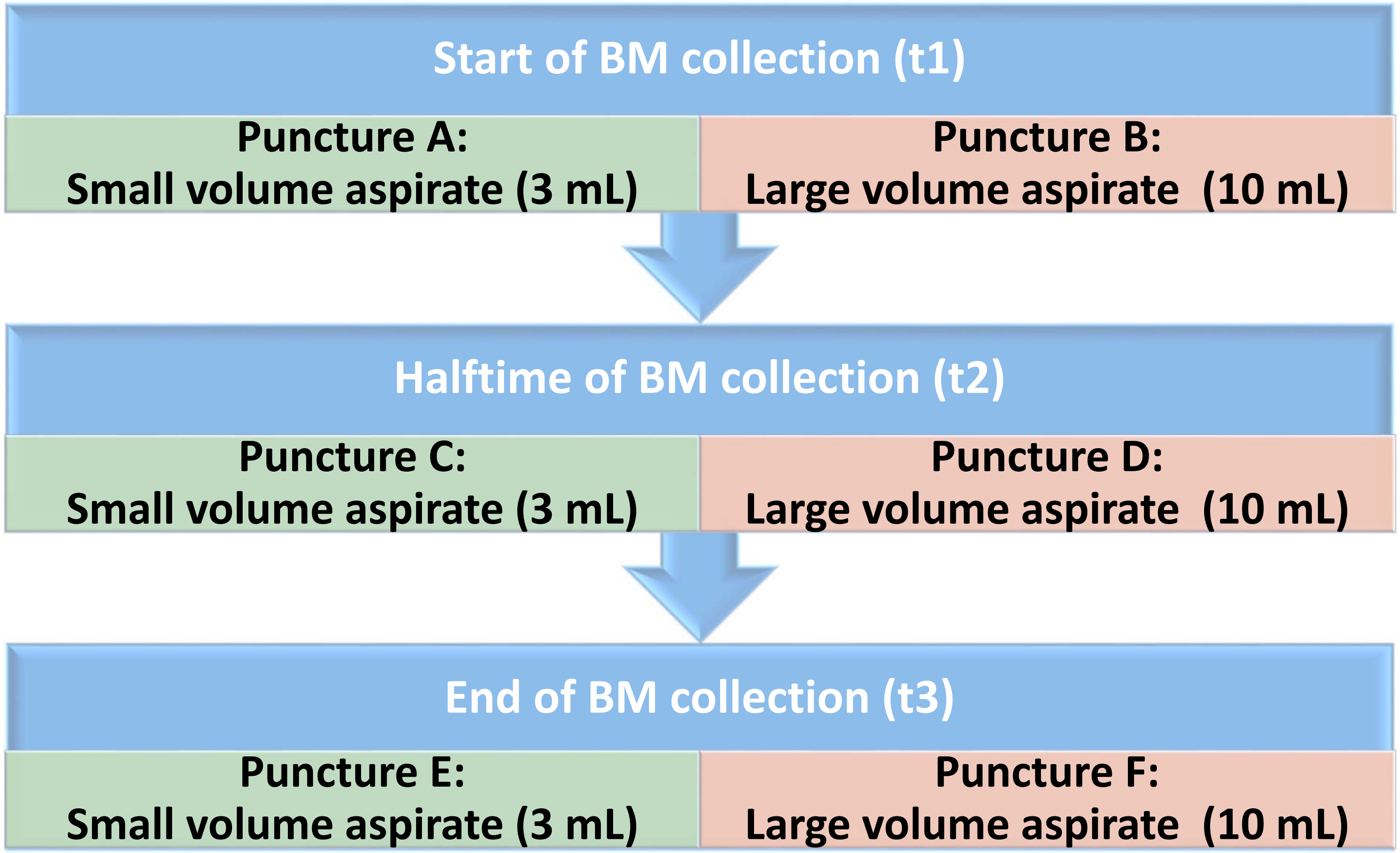

Additional file 1: Fig. S2

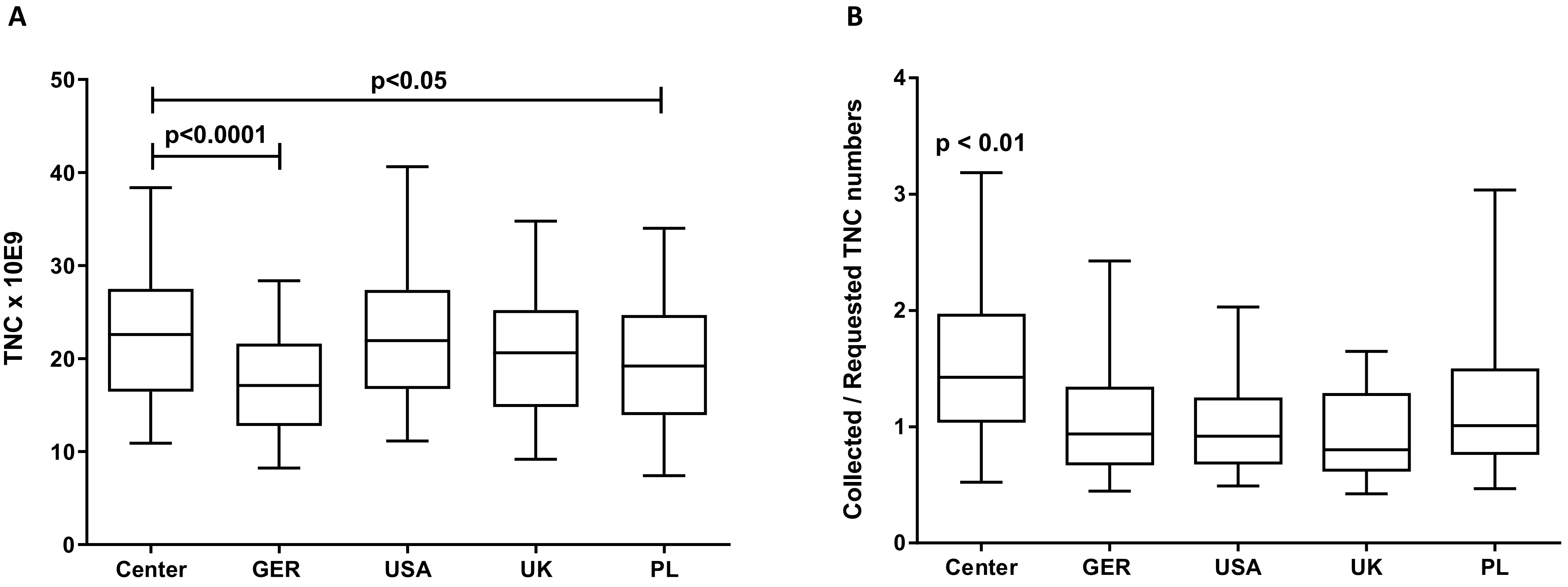

Additional file 1: Fig. S3

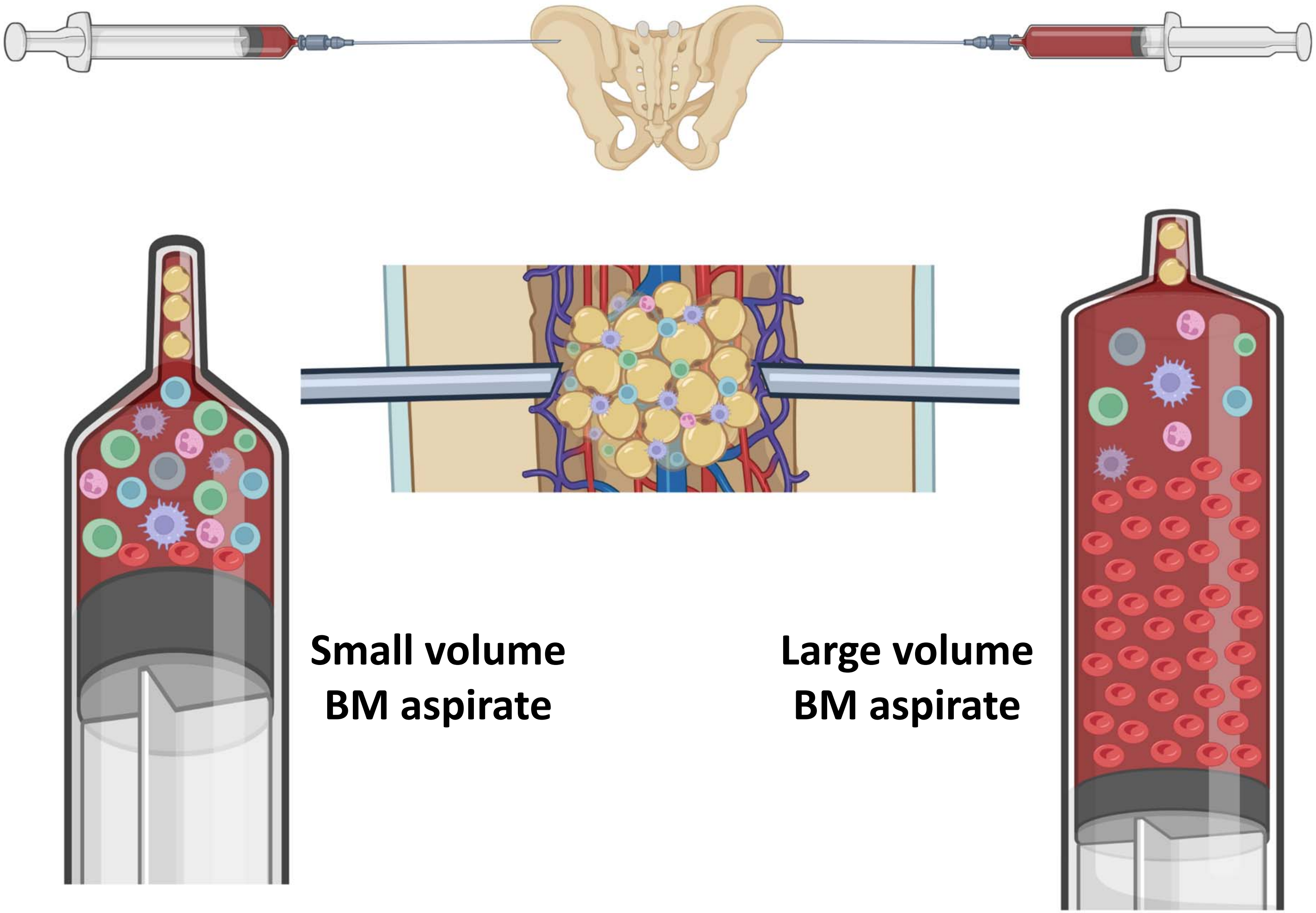

Additional file 1: Fig. S4

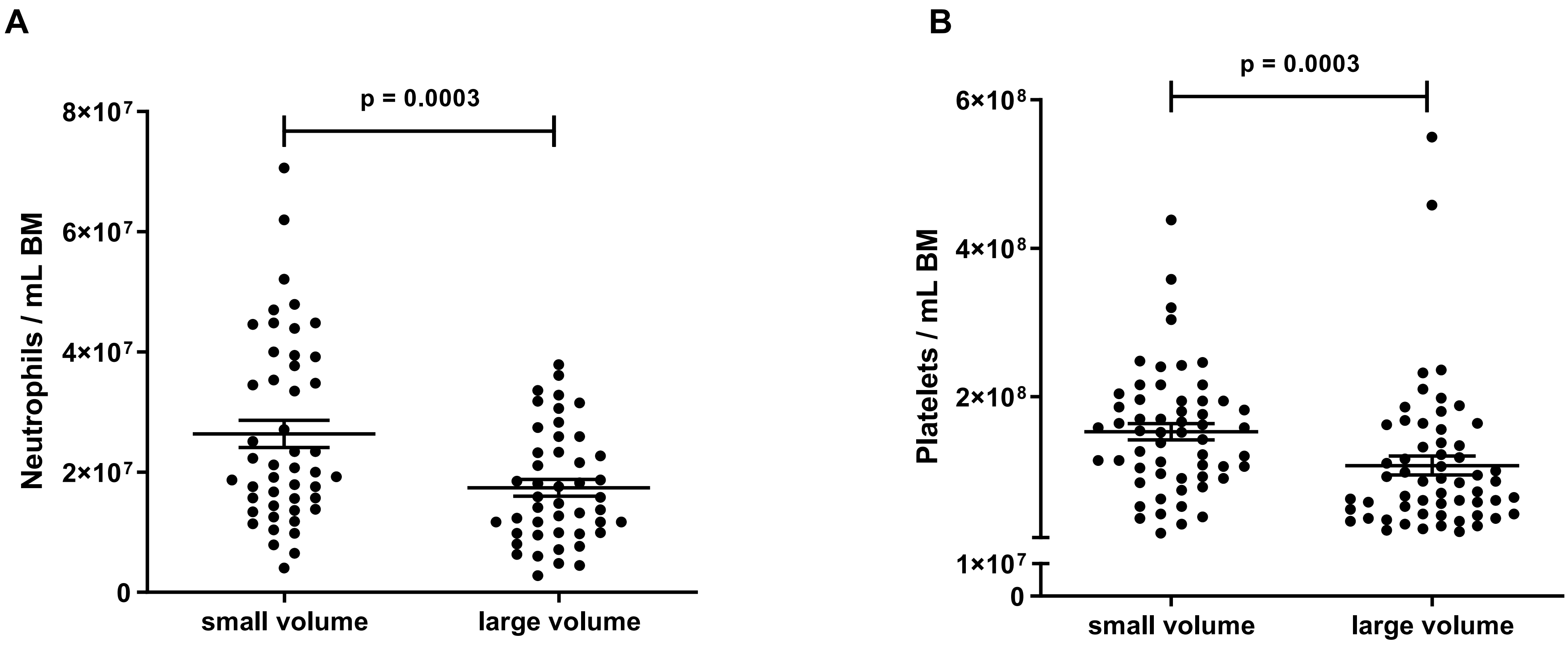

Additional file 1: Fig. S5

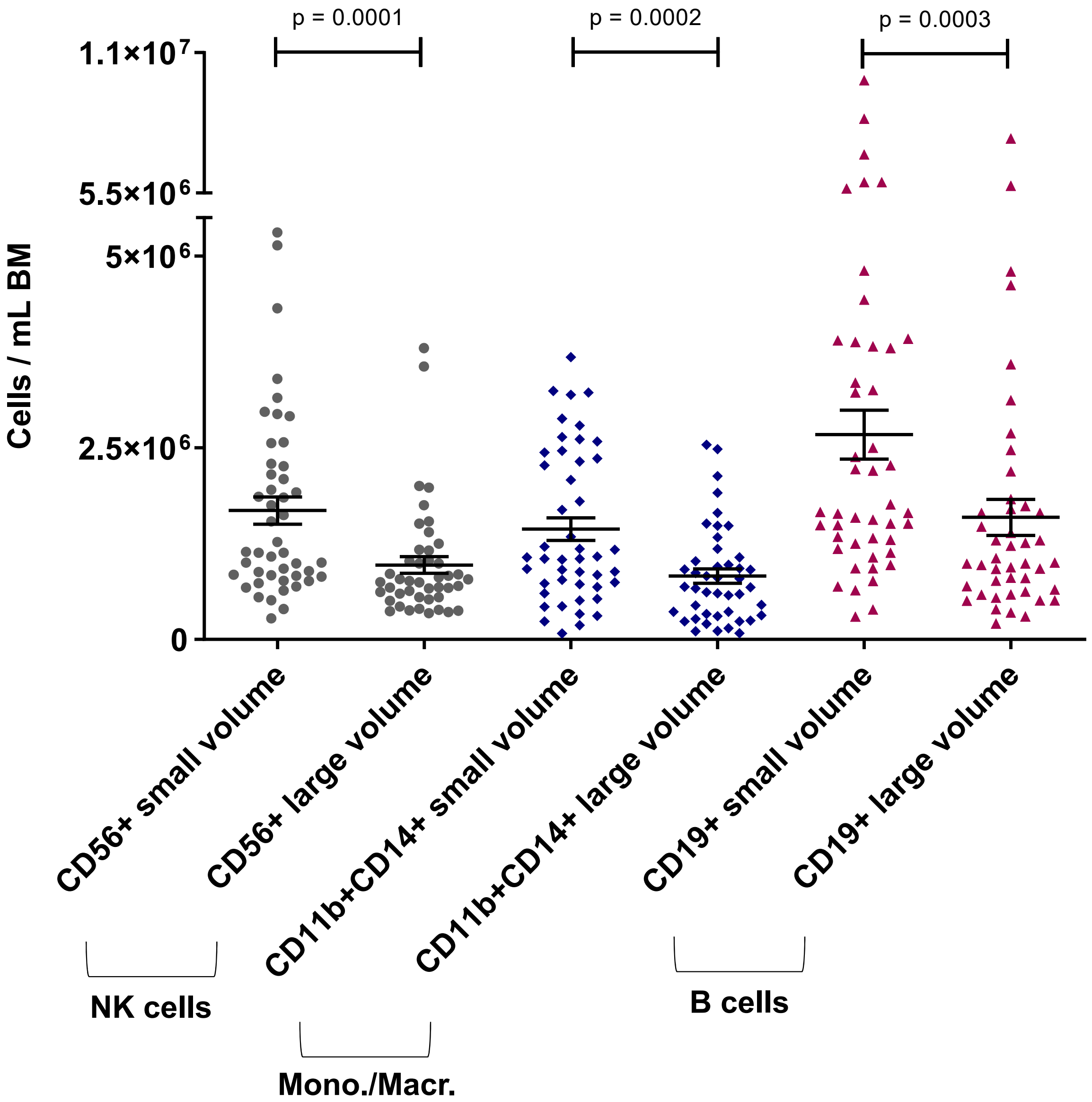

Additional file 1: Fig. S6

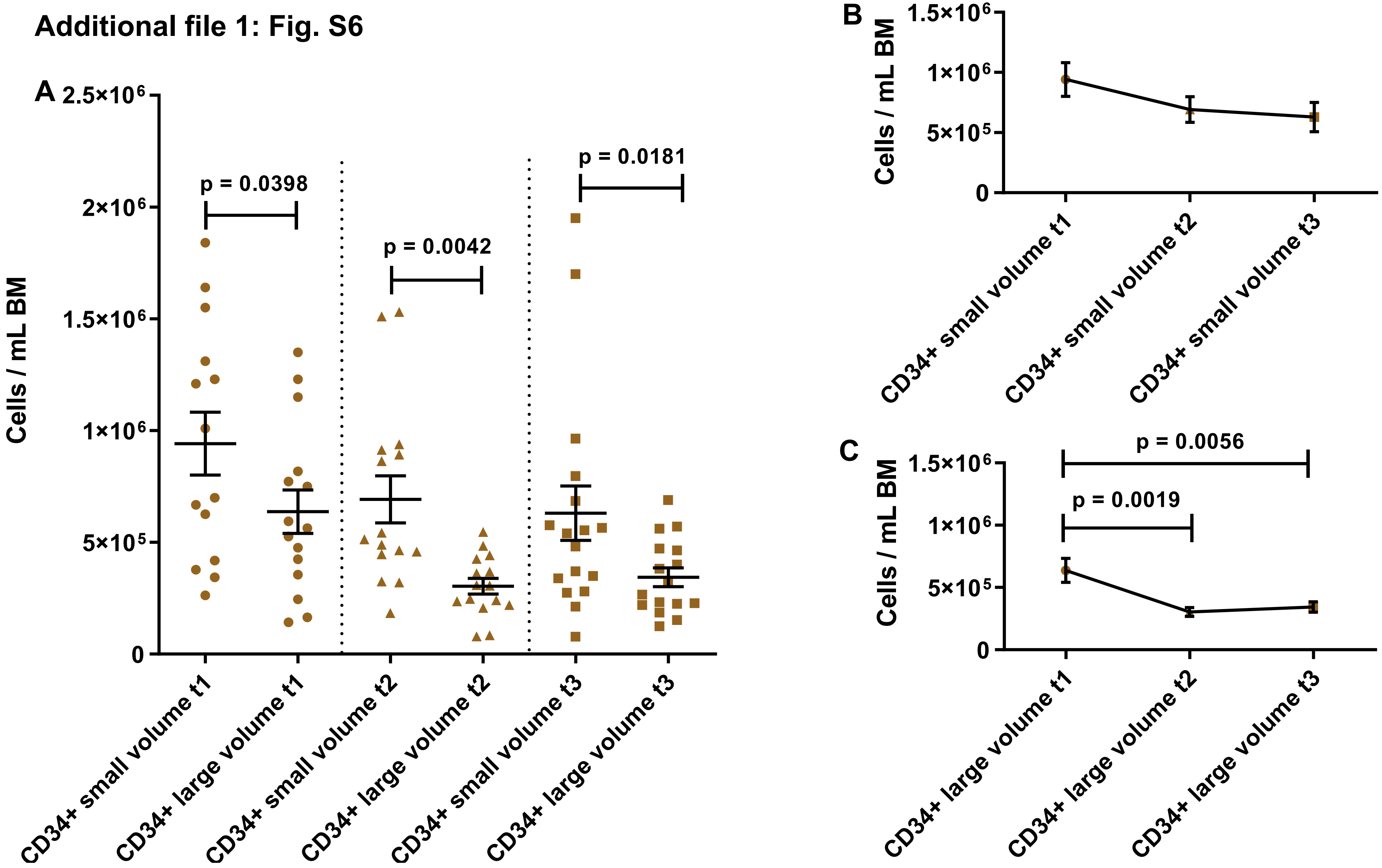

Additional file 1: Fig. S7

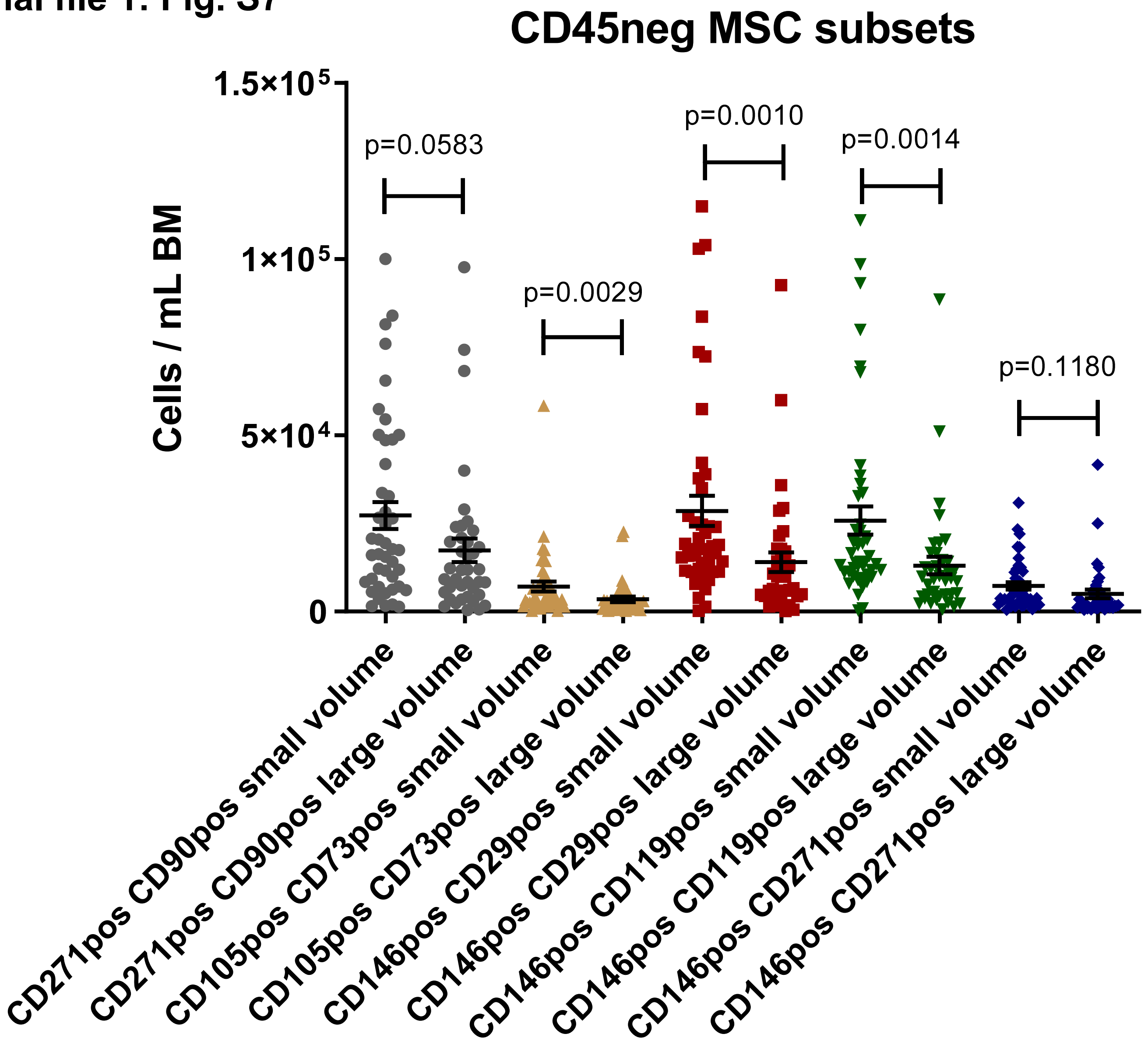

Additional file 1: Fig. S8

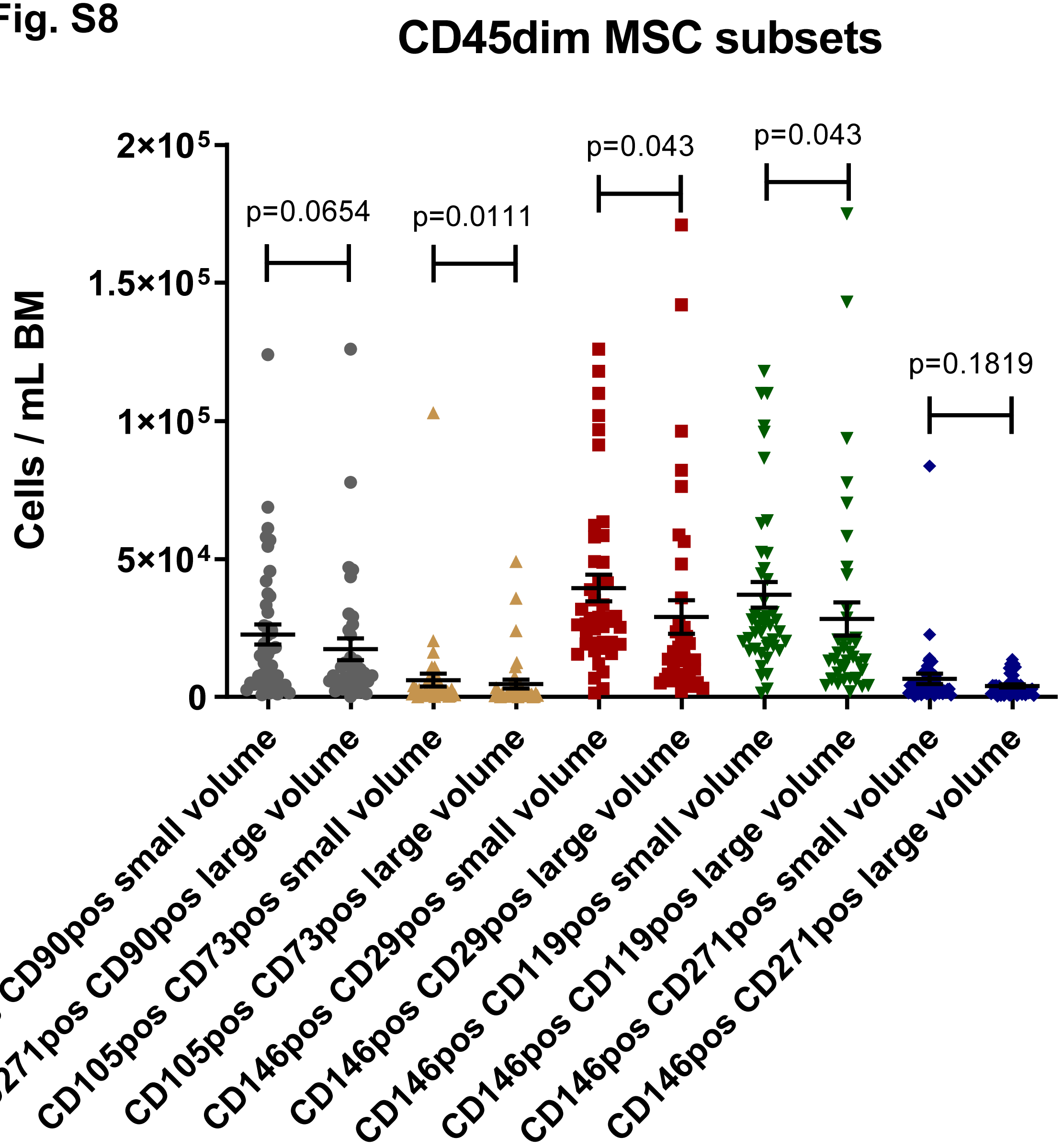

Additional file 1: Fig. S9

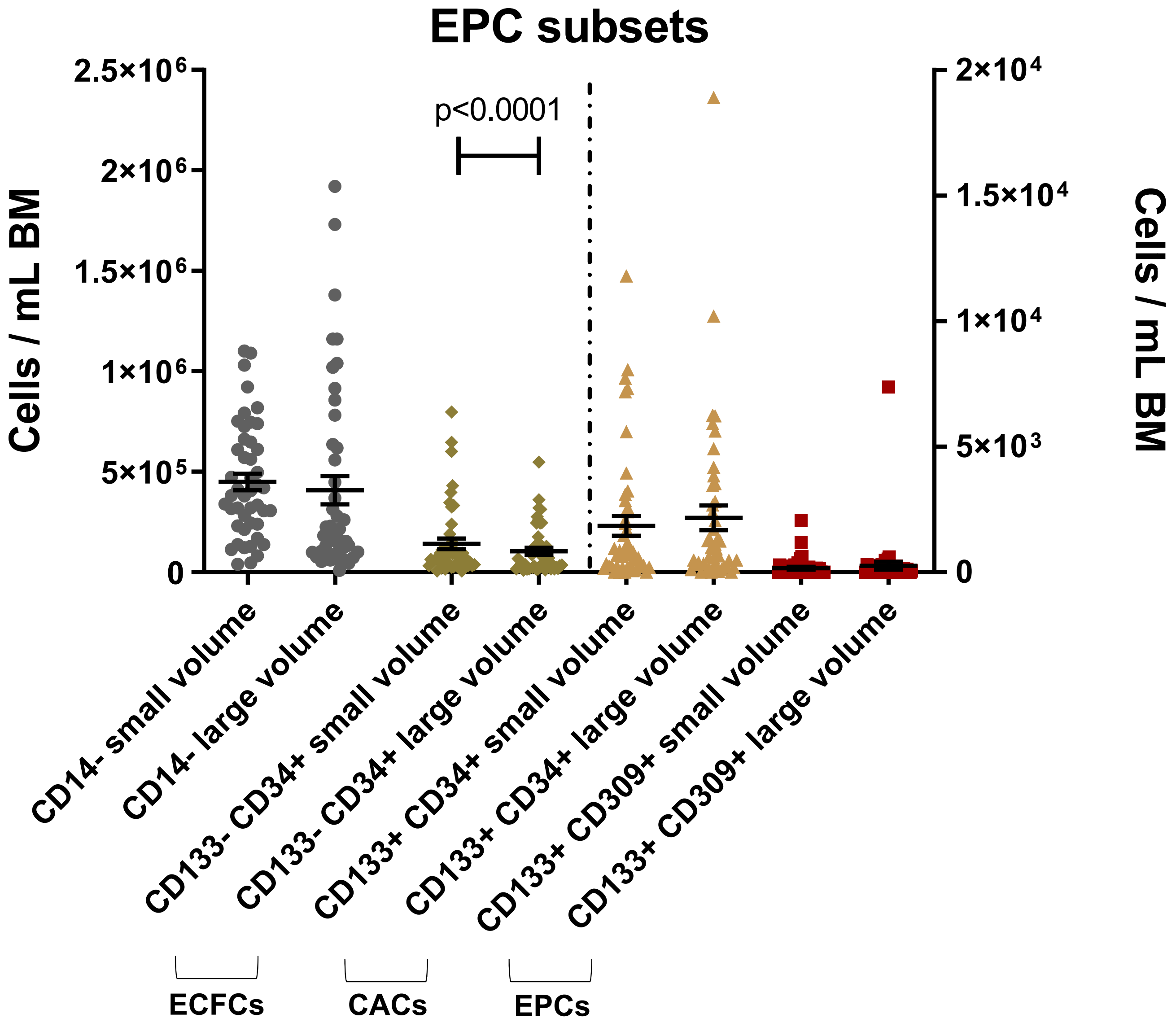

Additional file 1: Fig. S10

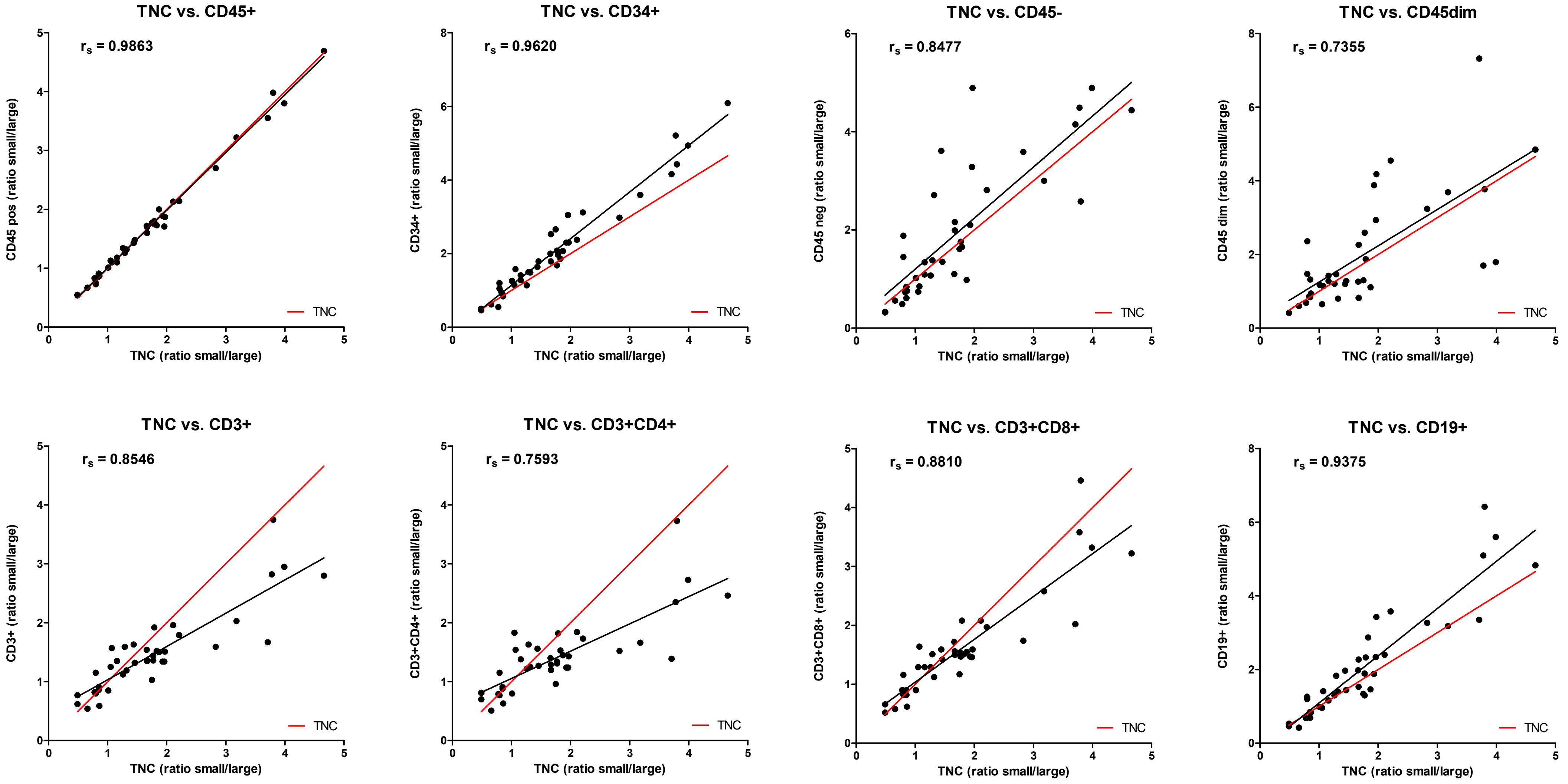

Additional file 1: Fig. S10 (continued)

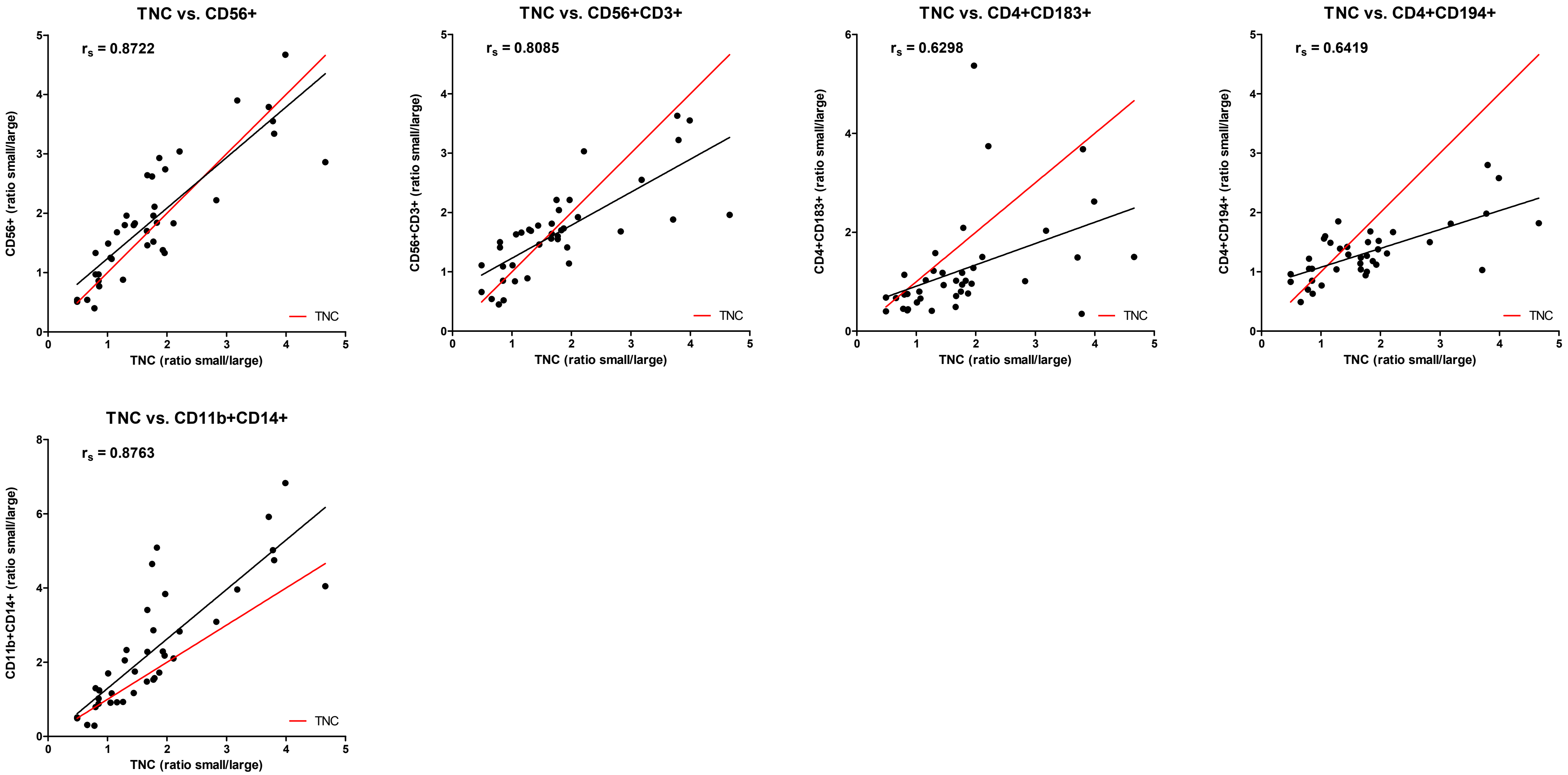

Additional file 1: Fig. S11

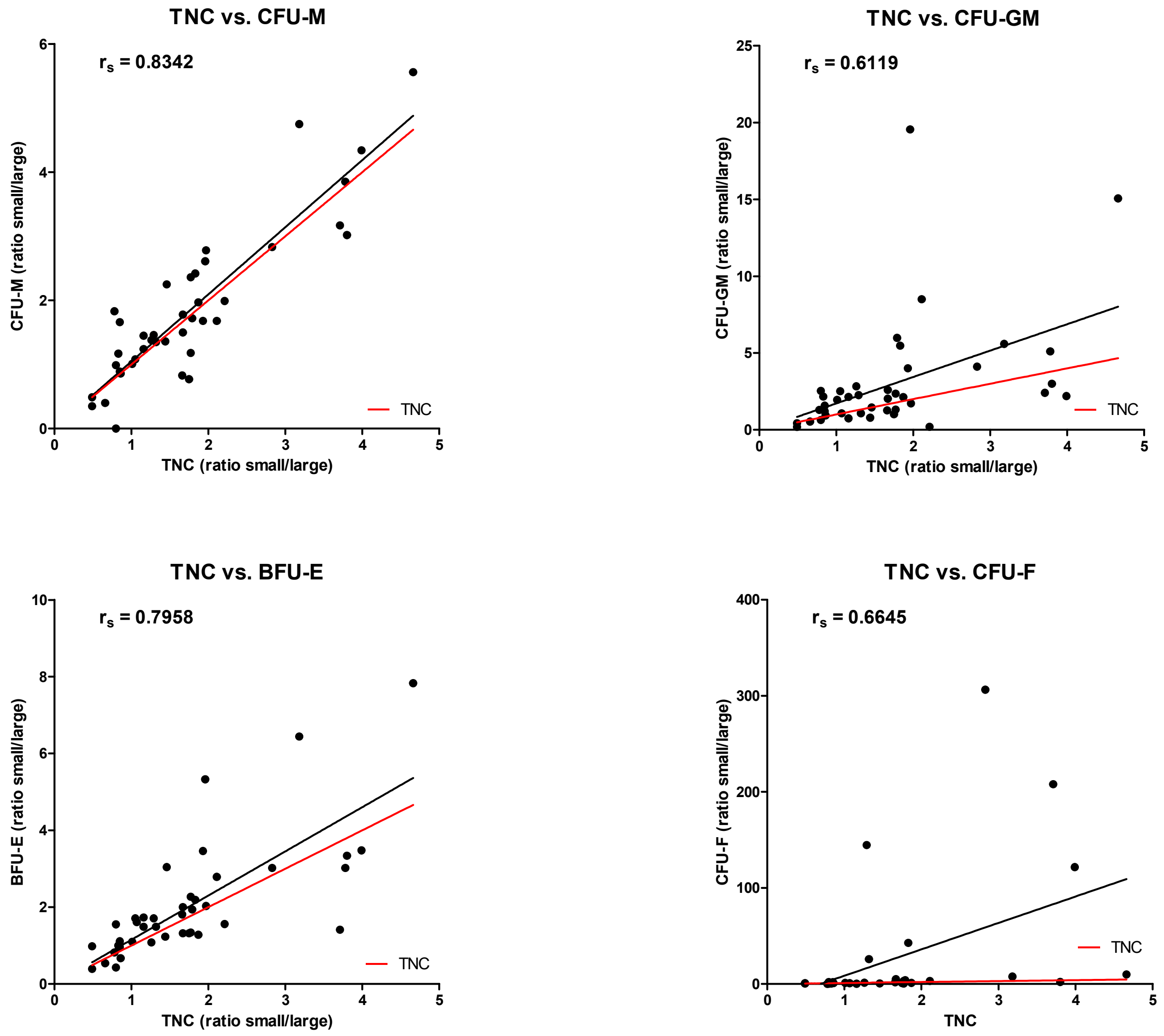

Additional file 1: Fig. S12

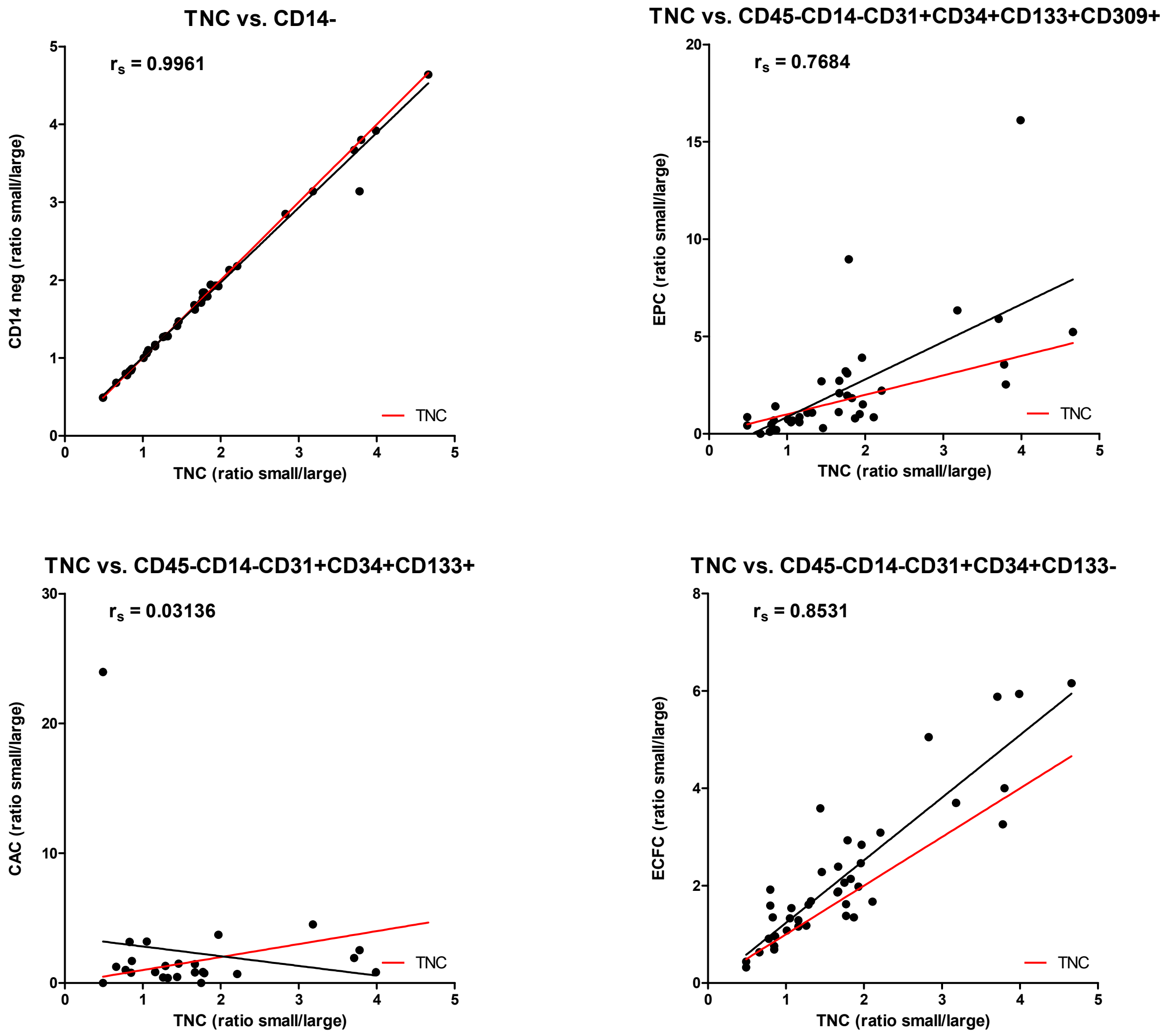

Supplement: Supplementary file 1 — Additional file 1: Fig. S1. Sampling overview. For each BM collection three small volume BM aspirates (Punctions A, C and E) as well as three large volume BM aspirates (Punctions B, D and F) were sampled at three time points. To standardize sampling and to exclude possible impact of the needle position in the punction canal each sample was taken from separate punctions as first aspirate after reaching the BM cavity. N = 20 donors. Fig. S2. Benchmarking HPC-M collection centers worldwide. A. TNC absolute numbers in HPC-M collected in our center (Center) and in other international collection centers. B. Ratio collected to requested TNC numbers in HPC-M collected in our center and in other international collection centers. One-way ANOVA (Kruskall-Wallis test) followed by Dunn’s multiple comparisons test; error bars: 5 - 95 percentile; N = 81 for our center (Center), N = 829 for GER (Germany without our center), N= 96 for USA, N = 33 for UK, N = 209 for PL. Fig. S3. Hypothesis. Visualization of the hypothesis that small volume BM aspirates are higher concentrated for progenitor cells compared to large volume BM aspirates that contain more red blood cells thus collecting fewer progenitor cells per milliliter. The main defining factor for the cell types being differentially collected is their specific gravity. Fig. S4. Neutrophils and platelets in small volume aspirates vs. large volume aspirates. A. Neutrophils per mL BM in small volume aspirates vs. large volume aspirates. B. Platelets per mL BM in small volume aspirates vs. large volume aspirates. Wilcoxon matched pairs test; dots: individual samples; horizontal lines: means; error bars: SEM; 20 donors, 1-3 time points, 2 volumes each; small volume samples: N = 47 (neutrophils), N = 57 (platelets); large volume samples: N = 46 (neutrophils), N = 58 (platelets). Fig. S5. Mature CD45pos immune cells in small volume aspirates vs. large volume aspirates. Wilcoxon matched pairs test; dots: individual samples; horizontal lin [file 12916_2023_3059_MOESM1_ESM.pdf]
